# Acoustically seeded fabrication of a DNA tesseract into a conductive wire

**DOI:** 10.1093/nar/gkaf1409

**Published:** 2025-12-31

**Authors:** Simon Chi-Chin Shiu, Marcello DeLuca, Wai Hin Chui, Pingping Zhang, Xiaoyong Mo, Ryan Ho Ping Siu, Erqian Dong, Sichao Qu, Calvin Chun Long Cheung, Andrew B Kinghorn, William L Whitehouse, Jingyu Cui, Weisi He, Xue-Yan Wang, Jiahui Li, Areebah Gul Khan, Sophie H von Torklus, Tsz Fai Yu, Khuloud T Al-Jamal, Edmund C M Tse, Gaurav Arya, Nicholas Xuanlai Fang, Keda Zhou, Julian A Tanner

**Affiliations:** School of Biomedical Sciences, Li Ka Shing Faculty of Medicine, The University of Hong Kong, Hong Kong, P.R. China; Department of Mechanical Engineering and Materials Science, Duke University, Durham, NC 27708, United States; School of Biomedical Sciences, Li Ka Shing Faculty of Medicine, The University of Hong Kong, Hong Kong, P.R. China; Department of Mechanical Engineering, Faculty of Engineering, The University of Hong Kong, Hong Kong, P.R. China; Department of Chemistry, HKU-CAS Joint Laboratory on New Materials, Faculty of Science, The University of Hong Kong, Hong Kong, P.R. China; Laboratory for Synthetic Chemistry and Chemical Biology Limited, Units 1503-1511, 15/F, Building 17W, Hong Kong Science Park, New Territories, Hong Kong, P.R. China; School of Biomedical Sciences, Li Ka Shing Faculty of Medicine, The University of Hong Kong, Hong Kong, P.R. China; Department of Mechanical Engineering, Faculty of Engineering, The University of Hong Kong, Hong Kong, P.R. China; Materials Innovation Institute for Life Sciences and Energy (MILES), HKU-SIRI, Shenzhen 518000, P.R. China; Department of Mechanical Engineering, Faculty of Engineering, The University of Hong Kong, Hong Kong, P.R. China; JC STEM Lab of Nanomedicine for Advanced Therapy, Department of Pharmacology and Pharmacy, Li Ka Shing Faculty of Medicine, The University of Hong Kong, Hong Kong, P.R. China; School of Biomedical Sciences, Li Ka Shing Faculty of Medicine, The University of Hong Kong, Hong Kong, P.R. China; Advanced Biomedical Instrumentation Centre, Hong Kong Science Park, Shatin, New Territories, Hong Kong, P.R. China; School of Biomedical Sciences, Li Ka Shing Faculty of Medicine, The University of Hong Kong, Hong Kong, P.R. China; School of Biomedical Sciences, Li Ka Shing Faculty of Medicine, The University of Hong Kong, Hong Kong, P.R. China; School of Biomedical Sciences, Li Ka Shing Faculty of Medicine, The University of Hong Kong, Hong Kong, P.R. China; School of Biomedical Sciences, Li Ka Shing Faculty of Medicine, The University of Hong Kong, Hong Kong, P.R. China; School of Biomedical Sciences, Li Ka Shing Faculty of Medicine, The University of Hong Kong, Hong Kong, P.R. China; Department of Biochemistry, University of Oxford, Oxford, OX1 2JD, United Kingdom; Department of Mechanical Engineering, Faculty of Engineering, The University of Hong Kong, Hong Kong, P.R. China; JC STEM Lab of Nanomedicine for Advanced Therapy, Department of Pharmacology and Pharmacy, Li Ka Shing Faculty of Medicine, The University of Hong Kong, Hong Kong, P.R. China; Department of Chemistry, HKU-CAS Joint Laboratory on New Materials, Faculty of Science, The University of Hong Kong, Hong Kong, P.R. China; Laboratory for Synthetic Chemistry and Chemical Biology Limited, Units 1503-1511, 15/F, Building 17W, Hong Kong Science Park, New Territories, Hong Kong, P.R. China; Department of Mechanical Engineering and Materials Science, Duke University, Durham, NC 27708, United States; Department of Mechanical Engineering, Faculty of Engineering, The University of Hong Kong, Hong Kong, P.R. China; Materials Innovation Institute for Life Sciences and Energy (MILES), HKU-SIRI, Shenzhen 518000, P.R. China; School of Biomedical Sciences, Li Ka Shing Faculty of Medicine, The University of Hong Kong, Hong Kong, P.R. China; School of Biomedical Sciences, Li Ka Shing Faculty of Medicine, The University of Hong Kong, Hong Kong, P.R. China; Materials Innovation Institute for Life Sciences and Energy (MILES), HKU-SIRI, Shenzhen 518000, P.R. China; Advanced Biomedical Instrumentation Centre, Hong Kong Science Park, Shatin, New Territories, Hong Kong, P.R. China; School of Biomedical Engineering, The University of Hong Kong, Hong Kong, P.R. China

## Abstract

Assembly of DNA nanostructures to sub-millimetre scales is expected to have significant potential for applications in materials science and medicine. One approach to control nanostructure growth is through using acoustic waves to create pressure nodes for clustering. Here, we report a facet-based underlying DNA nanostructure architecture with structural and stability characteristics ideal for acoustic patterning. The architecture comprises only 16 canonical DNA oligonucleotides which self-assemble to form a nested cube, inspired by the four-dimensional hypercube known as a “tesseract.” Cryogenic electron microscopy (Cryo-EM) and atomic force microscopy (AFM) analysis revealed a fully formed tesseract structure with exceptional stiffness and a melting temperature of 84°C, significantly higher than other unmodified DNA nanostructures. The DNA tesseract nanostructures could be acoustically shaped into wires spanning over 500 µm, observed after deposition onto an interdigitated electrode (IDE). The wires were shown to be electrically conductive, highlighting unique prospects for application. Simplified bottom-up assembly of a small number of oligonucleotides into a relatively complex and structurally stable DNA nanostructure with characteristics ideal for modular assembly holds promise for applications across bioelectronics and other fields.

## Graphical Abstract

**Graphical Abstract:**
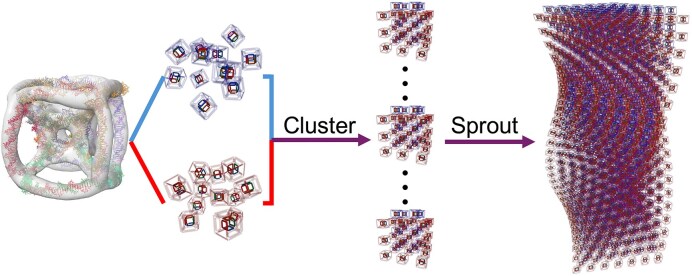

## Introduction

Micron-scale assembly of metamaterials allows for new possibilities in bioelectronics, which is especially important for rapidly developing areas of technology such as wearable devices [1, 2]. Biocompatibility is often one of the major concerns for these devices and DNA is theoretically amongst the most compatible materials for such purpose. In DNA nanotechnology, large scale assembly is often achieved by complementary overhang sequences on individual units for polymerization [3–5]. Growth typically relies on the design of overhangs and assembly conditions without further directional control. However, extending assembly control to the micron scale could be important for fabricating DNA into an actual device. Given the capability of DNA in conducting electricity [6–8], one innovative approach is using surface acoustic wave (SAW) to direct DNA origamis that could result in a linear stream of crystals of over 100 µm [9]. Yet, existing DNA nanostructures are often thermally unstable (melting temperatures: 50–70°C) [10–13] and require sophisticated design or exotic modifications for the structural stability to withstand shear forces and localized heating [14–16]. In this work, we investigate the tesseract structure as a possible solution. The tesseract is a four-dimensional hypercube considered as the natural successor to the two-dimensional square and three-dimensional cube [17, 18]. In chemistry and materials science, a fully designable, self-encapsulating, and modular tesseract structure has not been synthesized, neither as a hydrocarbon framework nor as a nanostructure. Theoretical studies have predicted significant thermal and kinetic stability and potentially unusual biophysical properties of molecules with such architecture [19]. Inorganic tesseracts have been etched from transition metal alloys [20] but would be difficult to design and scale for various applications or other functionalities [21, 22]. A nested cube with a similar shape as the tesseract has been made using a DNA origami strategy [10]. However, such a DNA origami approach uses a long phage DNA scaffold coupled to ∼100 smaller staple oligonucleotides with multiple single stranded breaks, unsuitable for the exceptional stability required for acoustic shaping.

**Figure 1.**
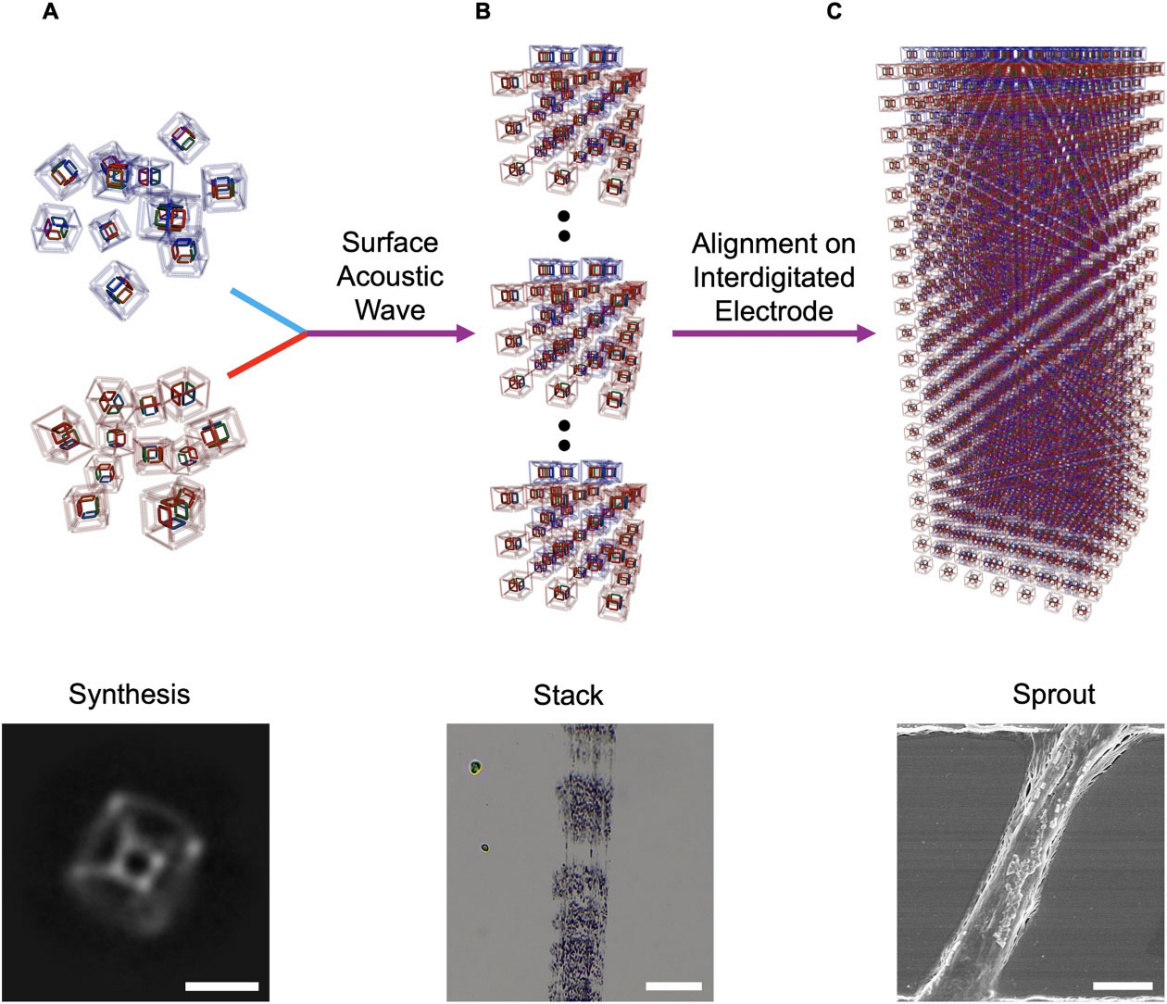
Three stages of bottom-up assembly of a DNA tesseract wire. Suggested mechanism of microscale assembly of the DNA tesseract into a long wire. DNA tesseracts were assembled separately with complementary anchors (**A**) and mixed after concentration. The image at the bottom shows the front angle of the DNA tesseract as one of the 2D classifications from Cryo-EM; scale bar: 5 nm. (**B**) The aligned clusters of DNA tesseract in capillaries; scale bar: 20 µm. The clusters were pushed out of the capillaries and deposited onto IDE pretreated with thiolated anchors that were complementary to the overhangs on the tesseract. The droplet was incubated on IDE overnight for the wire formation (**C**); scale bar: 2 µm.

Herein, we report the design and structural characterization of a DNA tesseract (a nested cubic framework) built from just 16 oligonucleotides with remarkable mechanical properties and thermal stability. The tesseract’s mechanical robustness enables its alignment into micron-scale conductive wires via SAW patterning, overcoming a critical barrier to functional DNA-based electronics. We then use this DNA tesseract to acoustically shape a micron-scale conductive wire as shown in Fig. 1. We first developed a new facet-based design approach, which requires only 16 DNA oligonucleotides in equimolar ratio, rather than hundreds of single strands in excess amount to scaffold for origami-based approaches [23]. The simple assembly method has high assembly efficiency even without purification under optimal DNA concentration conditions. The DNA tesseract also shows high thermal stability with a melting temperature of 84°C. Cryo-EM and AFM reveal the expected hypercube structure with significant structural rigidity. Thermal, spatial and mechanical stabilities were conferred to an encapsulated small cube through the facet-based design of tesseract. The resulting DNA tesseract combines unprecedented thermal stability and mechanically reinforced inner cube, surpassing most unmodified DNA nanostructures. The stability allows the aggregation into clusters by application of SAW. The clusters of tesseracts were found to grow into a long wire when deposited onto an IDE and were found to be more conductive than before the application of SAW. Remarkably, SAW-directed growth produces sub-millimeter conductive wires with a 4.21-fold increase in relative conductivity compared to randomly aggregated tesseracts, demonstrating the first acoustically shaped DNA-based wire.

## Materials and methods

### Design and assembly of DNA tesseract

The DNA tesseract was designed using Tiamat 2.0 [24]. Each of the four components (A, B, Bc, and Sc) consists of four single-stranded DNA occupying one face of the structure. Equimolar concentrations of single-stranded DNAs (Integrated DNA Technologies and Sangon, nanodrop corrected to 10 µM) were mixed in deionized water to make up a 2.5 µM working solution of the individual components. Equimolar concentrations of the working solutions were mixed in 1× phosphate buffer saline (Sigma; 140 mM NaCl, 3 mM KCl, 10 mM phosphate buffer) at a final concentration of 20 nM for assembly. For gel electrophoresis, samples were prepared in 1× TAEM (40 mM Tris-acetate, 1 mM EDTA, and 12.5 mM magnesium acetate). The assembly mixture was annealed in a thermal cycler (Applied Biosystems ProFlex PCR System). The mixture was incubated at 95°C for 3 min then slowly annealed from 95°C to 20°C at the rate of 4.4°C/h. The folded structures were stored at room temperature.

### Gel electrophoresis

For agarose gel electrophoresis, 2.5% agarose gel in 1× TAEM was used to observe the migration of DNA tesseract and the partial assemblies. For polyacrylamide gel electrophoresis (PAGE), it was only used to observe the assembly of small cube. 15% native PAGE in 1× TAEM was used for the experiment. All gel electrophoresis were performed at 65 V at 4°C. Four hours were used for agarose gel electrophoresis and one hour was used for the PAGE. The fluorescent signal from the agarose gel electrophoresis was observed with Analytical GE Amersham Typhoon5 Biomolecular Imager (photomultiplier tube potential: 322 V). For gels stained with SYBR gold (Invitrogen), prestain was done by mixing 10 µl of 20 nM sample with 2 µl loading mixture (5× SYBR gold and 4× loading dye). Signal was observed with ChemiDoc (Bio-Rad).

### Quantitative fluorescent measurement

20 nM DNA tesseract and partial assemblies were assembled in 1× Phosphate Buffer Saline (PBS) (strands on small cube were labeled with Cy3 and Cy5). The fluorescent signals of fluorescent resonance energy transfer (FRET) (excitation: 550 nm, emission: 668 nm) were measured using Thermo Varioskan Flash microplate reader. Bandwidth was 5 nm and measurement time was 500 ms.

### Melting analysis in quantitative polymerase chain reaction (qPCR)

For qPCR, 100 nM DNA tesseract and partial assemblies were assembled in 1× PBS (concentration determined in Supplementary Fig. S11). SYBR green was used to observe the presence of duplex DNA in the mixture with ROX as a normalising control. The samples were subjected to melting from 20°C to 95°C at the rate of 0.5°C/min in Bio-Rad Opus 96. The derivatives of raw data were calculated to find out the melting temperature.

### Circular dichroism

For circular dichroism (CD), 300 nM DNA tesseract and partial assemblies were assembled for CD using Jasco J-1500. 600 µl of sample was transferred to a quartz cuvette with 1 cm pathlength. Five repetitive measurements were done by scanning the spectrum from 220 to 320 nm with 1 nm bandwidth. Data pitch was 1 nm and data integration time were 2 s. To observe melting, measurement was repeated for every 5°C from 20°C to 95°C. The obtained spectra were smoothed using Savitsky–Golay filter in Origin with polynomial order of 4 and a smoothing window of 15 points. The peak values from 247 and 277 nm were obtained to calculate the derivatives along the temperature change.

### Dynamic light scattering

Particle size and size distribution of the DNA tesseract were characterized by dynamic light scattering (DLS), using the Zetasizer Pro particle size analyzer with a 4.0 mW He-Ne laser at a detection angle of 173°, and the ZS Xplorer software (Malvern Panalytical Ltd., Worcestershire, UK). Samples were dispersed in 1× PBS, loaded in a low-volume quartz cuvette, and measured in triplicates.

### Atomic force microscopy

The acquisition of AFM images was performed using a Bruker NanoWizard ULTRA Speed 2 AFM, employing the peakforce tapping mode in a fluidic environment. SNL-10d AFM tapping mode probes (Bruker Nano, Inc.) with an average force constant of 0.06 N/m and a mean resonance frequency of 18 kHz was used in imaging with scan areas of 1 μm × 1 μm. A scanning resolution of 256 lines with 256 pixels per line with a scan rate of 1 Hz was maintained throughout all image acquisitions. The JPK Data Processing software program was utilized for the meticulous analysis of DNA contour lengths, as previously described. The AFM and force–distance curve measurements were carried out in 1× TAE buffer with 7 mM NiCl_2_ and analyzed following a published protocol. To obtain Young’s modulus (*E*) value, the deflection sensitivity of the probe was calibrated on the mica surface several times and the mean value was taken. The spring constant of the probe was determined using the Thermal Tune function of the AFM software. The Young’s modulus (*E*) value was determined by fitting the data to the Sneddon (conical indenter) model, where *F* = force (from force curve), *E* = Young’s modulus (fit parameter), *ν* = Poisson’s ratio (sample dependent, typically 0.2–0.5, for the DNA Tesseract, the Poisson coefficient should be ~0.3), α = half-angle of the indenter (20 degree), and δ = indentation.

### Cryo-EM sample preparation, data collection, and analysis

The assembled DNA tesseract at 20 nM in PBS was prepared in sufficient amount to be concentrated using Amicron centrifugal filter with 100 kDa cutoff. Centrifugation was performed at 14 000 × *g* for 10 min at room temperature. Multiple centrifugations were performed until the concentration of DNA tesseract reached 10 µM as confirmed by nanodrop. After concentration, 3.5 µl of concentrated DNA Tesseract was then applied to glow-discharged holey carbon grid (Quantifoil 1.2/1.3) and vitrified using Vitrobot Mark IV (ThermoFisher Scientific) at 4°C, 100% humidity, 0 s wait time, 3 s blot time, and 0 blot force. The DNA tesseract was imaged at nominal magnification of ×130 000 on a 300 kV FEI Titan Krios. Pixel size was 0.9557 Å. Movies were captured in counting mode with electron dose rate at 50 eÅ^−2^ using the EPU software and Falcon 3 detector (FEI). Defocus range was −1.2 to −2.6 µm. The raw movies were motion corrected, followed by constant transfer function (CTF) estimation in CryoSPARC 4.4. Particles were manually picked as template for Topaz training to generate a picking model. The model was used to automatically pick particles from the entire movie set. A sub-set of the picks was inspected and corrected as a new template for another round of training. Three Topaz trainings were performed in the same manner for auto-picking the dataset. 2D and 3D classification was performed to select intact particle for the reconstruction. A total of 4745 particles were selected to reconstruct a 3D map using the *ab initio* model with C1 symmetry. To enhance the resolution of the 3D map, octahedral symmetry was applied for the reconstruction again as well as the non-uniform refinement. Half-maps and full maps were submitted to 3DFSC processing server for validating the final resolution [25]. The illustrations of refined maps and 3D resolution maps were performed in ChimeraX [26].

### Coarse-grained molecular dynamics simulations

The tesseract and all sub-assemblies were simulated using the oxDNA coarse-grained model at 298 K and an effective monovalent salt concentration of 0.5 M (in essence accounting for both monovalent and divalent ions typical of DNA nanostructure fabrication conditions) [27]. A timestep of 15 fs was used for time integration. Mutual traps enforcing idealized base pairs were employed during relaxation stages but removed for the final production stage. Structures were simulated for 50–100 µs in the production stage, and mean structures were obtained using the structure averaging utility in the oxDNA analysis tools package [28].

### Surface acoustic wave for alignment of DNA tesseract

DNA tesseract, BcA or tetrahedron with two different set of complementary overhangs were assembled separately at 20 nM in 1× PBS. The products were concentrated to 200 nM and mixed for another slow annealing from 50°C to 20°C over 2 days. Samples were then transferred into hollow rectangle capillaries (CM Scientific, 0.05 mm × 1.00 mm × 50 mm) through capillary action and sealed with nail polish.

### SAW device fabrication

Interdigital transducers (IDTs) were patterned on a Y-cut lithium niobate (LiNbO₃) substrate via photolithography, with a 1500 μm gap between transmitter and receiver IDTs. Each IDT comprised 40 finger pairs of titanium/aluminum electrodes (2.5 μm width and 210 nm thickness), generating SAWs with a 10 μm wavelength at 342 MHz. The IDTs were wire-bonded to a printed circuit board (PCB).

### Acoustic alignment and deposition

The capillary was mounted between the IDTs on the LiNbO₃ substrate, with a thin layer of immersion oil (Nikon, Type A) applied to enhance acoustic coupling. A sinusoidal signal (342 MHz, 2 Vpp) was delivered to the transmitter IDT using a function generator (Siglent SDG 7102A), pulsed at 3 ms duration (1 000 000 cycles) with 27 ms intervals to mitigate thermal drift. Standing waves formed via interference between incident and reflected waves, concentrating nanostructures at pressure nodes within 30 min.

After applying SAW, one end of the capillary was cut, inserted into a cut gel loading tip and sealed with polydimethylsiloxane (PDMS). After the PDMS was dried, the other end of the capillary was cut, and the solution was pushed out with a pipette.

### Electrode biofunctionalization and tesseract wire hybridization

MicruX interdigitated gold electrodes (ED-IDE1-Au) were first rinsed thoroughly with 95% ethanol and MilliQ water. After drying, 10 µl of 0.5 M H_2_SO_4_ was dropped onto the electrode. The gold surface was activated by cyclic voltammetry (CV) scanning from −1 V to 1.3 V at 0.1 V/s for 12 cycles. The electrodes were rinsed again by MilliQ water and dried before DNA immobilization.

Thiol modified DNA anchor strands (sequences provided in Supplementary Table S1) were reconstituted to 100 µM and diluted to 10 µM in ultra-pure water. Then, the strands were reduced by Tris-(2-carboxyethyl) phosphine (TCEP; oligo to TCEP molar ratio was 1:100) for 2 h at room temperature (RT) to cleave the disulfide bonds. Reduced anchor strands were mixed and diluted to 0.5 µM each in 1× PBS. 10 µl of the mixture was dropped onto the activated gold surface and immobilized at RT overnight. After thorough washing with MiliQ water, the electrodes were blocked with 6 mM of mercaptohexanol (MCH) for 3 h at RT. Following the washing off unbound MCH, 5 µl of tesseract fiber sample was dropped onto the gold surface for strand hybridization. The reaction was performed at RT overnight. All incubation steps were conducted with humidity control to prevent solution drying.

### Electrical conductivity measurement

After fiber hybridization, the electrodes were rinsed briefly with 1× PBS and then blown dry. Linear sweep voltammetry (LSV) was performed using PalmSens 4 potentiostat (Houten, Netherlands). The LSV scan ranged from 0 to 0.8 V at a rate of 1 V/s. Electrical current readings were recorded at 0.1 V intervals. All electrochemical data were exported from the software PSTrace 5.9.

### AuNPs biofunctionalization and hybridization with the tesseract fiber on electrodes

Thiol modified DNA anchor strands (10 µM) were separately reduced by TCEP in water for 2 h at room temperature. Reduced anchor strands were mixed and diluted to 1 µM each in water for subsequent biofunctionalization.

1 ml of stock citrate capped AuNPs (741 957; Sigma) was centrifuged at 21 000 × *g* for 1 h at 4°C. After removing the supernatant, the AuNP pellet was resuspend into 100 µl of the mixed reduced DNA anchor strands for thiol conjugation. The suspension was incubated on an orbital shaker for 3 h at room temperature. NaCl was spiked into the suspension at a final concentration of 100 mM and continued to incubate at 4°C overnight with shaking. The AuNPs were pelleted again by centrifugation to remove the unbound oligo. Finally, the pellet was resuspended into 100 µl of 0.1× PBS and stored in 4°C.

Anchor strand immobilization efficiency on AuNPs was verified by 0.5% agarose gel electrophoresis in 1× TBE ran at 80 V for 30 min. The gel was imaged directly by Gel Documentation system without any staining.

After hybridizing the tesseract fiber onto the interdigitated electrodes (IDEs). The electrode surface was rinsed and dried. Then, 5 µl of AuNPs coated with the mixed anchor strands was dropped onto the electrode surface and incubated overnight at room temperature. Following rinsing in 1× PBS and airdrying the electrode surface, the LSV measurement was conducted again to characterize the effect of AuNPs patterning to changes in fiber conductivity.

### Scanning electron microscopy

Experiment was performed using Hitachi S-4800 field emission scanning electron microscope equips with a cold cathode field emission column emitter. The IDE with tesseract wire was sputter-coated with a 60:40 gold/palladium target using Quorum Q150T Plus ES for 40 s. Images were obtained with secondary electron at 5 kV accelerating voltage and sample distance of 14.2 mm. For energy dispersive X-ray spectroscopy, secondary electron at 20 kV and a X-Max 80 EDS detector were used with at least 150 000 counts Reports of element mapping were generated by AZtec software.

### Confocal microscopy

IDEs with observable wires under brightfield microscope were stained with 10 µl of 1 µM Cy3 anchor for 15 min. Fluorescence imaging was performed using a Zeiss LSM 900 inverted confocal microscope equipped with Airyscan 2. Imaging was conducted with a 20× objective, with excitation and emission at 548 nm and 561 nm (detection wavelength 569–617 nm) respectively. GaAsp-Pmt2 imaging device with GaAsP-PMT detector was used at 800 V detector gain. Effective numerical aperture was 0.8 and pinhole size was 122 µm. All images were processed using Zeiss ZEN Blue software for Airyscan reconstruction with only linear adjustments applied to brightness and contrast to preserve data integrity.

## Results

### Design of the DNA tesseract

**Figure 2.**
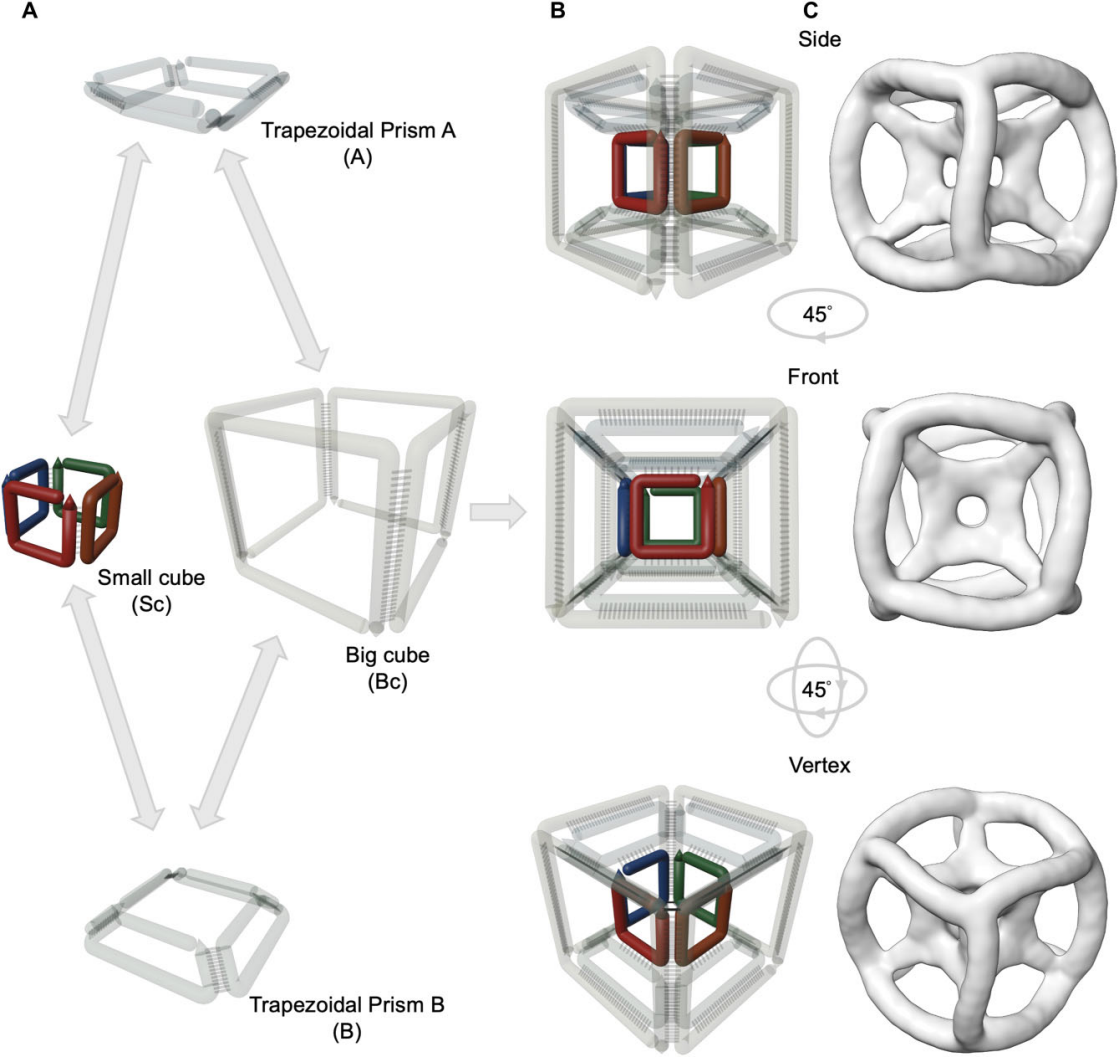
Design of the DNA tesseract. (**A**) Components of the DNA tesseract. Each sub-structure consists of four single-stranded DNA oligonucleotides that interact as indicated by the gray arrows. The small cube is encapsulated within the big cube through the two trapezoidal prisms. (**B**) Schematic of DNA tesseract from multiple perspectives. (**C**) 3D electron density maps of the DNA tesseract determined by Cryo-EM single particle analysis.

In order to synthesize a stable DNA tesseract, we used a facet-based design strategy using single-stranded DNAs [29]. As a three-dimensional projection of four dimensions, the tesseract is composed of a small cube within a big cube. The shape has four sub-structures (Fig. 2A): Big cube (Bc), Small cube (Sc) and two trapezoidal prisms termed “A” and “B.” Each sub-structure consists of four single-stranded DNAs with each strand making up one face of the sub-structure using a single thymidine at each vertex. These four sub-structures interact as depicted by the grey arrows in Fig. 2A. The small cube does not interact with the big cube directly but is captured within the tesseract through the presence of trapezoidal prisms. Fig. 2B shows the schematic diagrams of various perspectives of the tesseract. As shown in Fig. 2C, the structure has octahedral symmetry as confirmed by Cryo-EM.

### Formation of DNA tesseract

**Figure 3.**
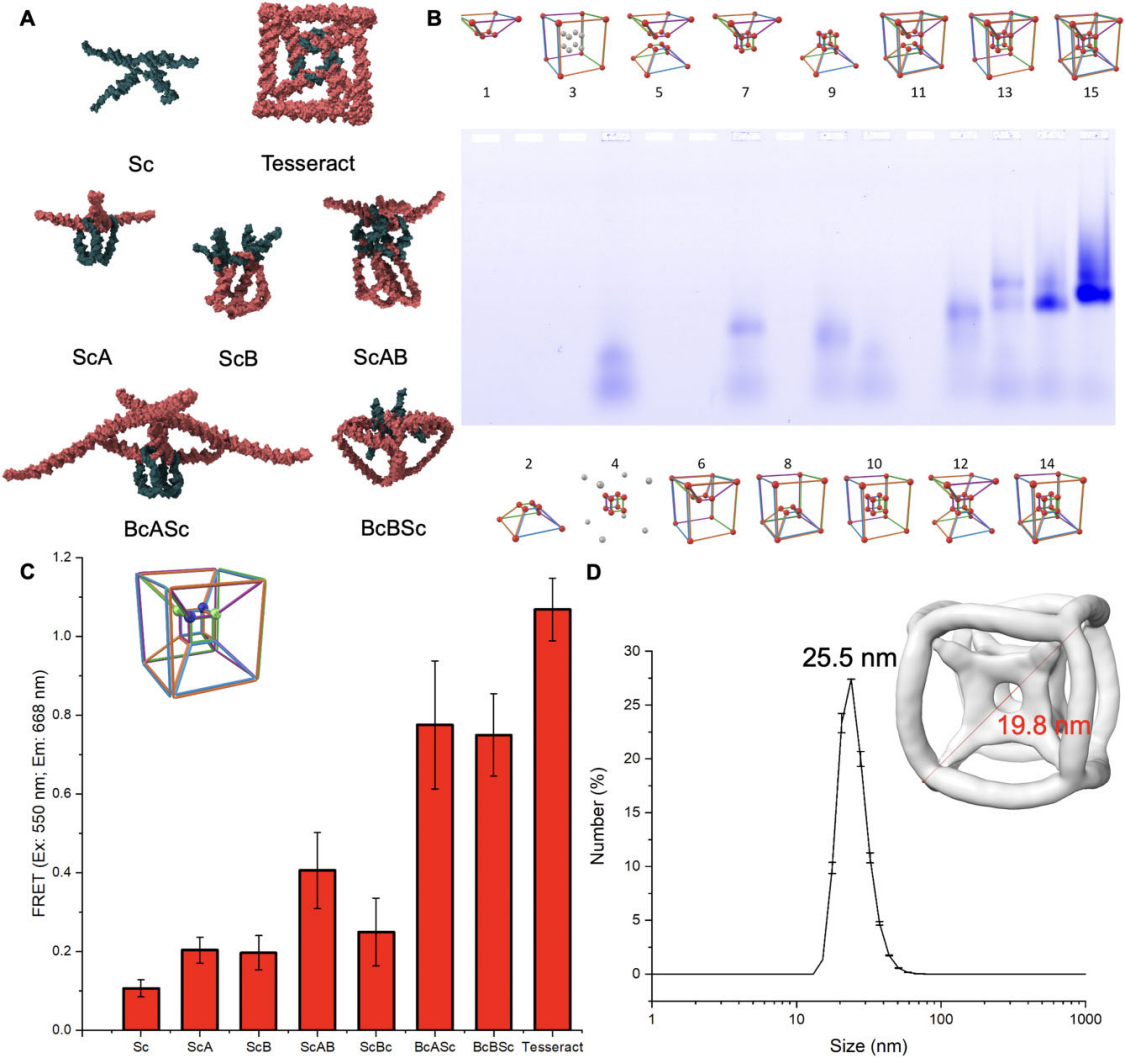
Partial assemblies of DNA tesseract components. (**A**) Simulated structure of small cube (Sc), DNA tesseract and other small cube binding components including big cube (Bc), Trapezoidal prism A and B (A, B). (**B**) Partial assemblies of DNA tesseract on agarose gel electrophoresis. Single-stranded DNAs for different combinations of components were mixed for assembly. The small cube was labeled with FRET pairs (two Cy3 and two Cy5). Lane 1: A; Lane 2: B; Lane 3: Bc; Lane 4: Sc; Lane 5: AB; Lane 6: BcA; Lane 7: ScA; Lane 8: BcB; Lane 9: ScB; Lane 10: BcSc; Lane 11: BcAB; Lane 12: ScAB; Lane 13: BcASc; Lane 14: BcBSc; Lane 15: BcABSc (tesseract). (**C**) FRET between 5′ ends of small cube in different combinations. Blue sphere: Cy3; Green sphere: Cy5. The stability of the small cube was progressively improved as it bound to more components of the tesseract as quantitatively measured. (**D**) Size characterization of the DNA tesseract. The mean hydrodynamic diameter of the tesseract measured by DLS was 25.5 nm. Inset shows the density map of the tesseract obtained from Cryo-EM with a measured body diagonal of 19.8 nm.

Before assembly experiments, we computationally tested the equilibrium structure of the tesseract design using oxDNA simulations. Figure 3A shows mean structures of the various sub-structure combinations obtained from simulations. Each sub-structure remained largely structureless in the absence of its complementary component, especially for the small cube, where nine base-pair complementarities between single strands were insufficient to realise proper assembly. When the small cube was bound to other sub-structures of the DNA tesseract, it assumed the cubic structure as designed. To experimentally observe the modular formation of the DNA tesseract we used an electrophoretic mobility shift assay (EMSA) to test the integrity of each assembled component (Supplementary Fig. S1). As predicted from the simulations, the big cube (Supplementary Fig. S1A) and both trapezoidal prisms (Supplementary Fig. S1C) formed higher-order structures, as observed by decreased gel migration, whereas the small cube did not form any higher structure (Supplementary Fig. S1B).

To further test the hypothesis that DNA tesseract assembly depends on stabilization of the outer big cube by a captured inner small cube, we assembled all 15 possible combinations of the four sub-structures of the tesseract and observed their migration via EMSA (Supplementary Fig. S1D). We observed slower migration as the complexity of combinations increased as well as 89.5% assembly efficiency (by comparing the band intensity of big cube on lane 3 and lane 15). However, we were not able to observe the Sc single strands due to the short length of DNA and contrast to other long sequences. Therefore, to enhance the sensitivity of detection for observing Sc, we labeled two strands of the Sc with Cy3 and the other two with Cy5 to facilitate a fluorescent resonance energy transfer (FRET) experiment (Fig. 3B). The same labels were in diagonal positions so that there were effectively four pairs available for FRET on the small cube. From the fluorescent EMSA, Sc single strands were observable with very weak FRET signal, whilst the other Sc combinations showed much stronger signal. Especially for the complete DNA tesseract, the FRET signal remained stable under prolonged electrophoresis, suggesting high stability of the complete structure (Supplementary Fig. S2). This suggested a possibility that the formation of small cube was stabilized when bound to other sub-structures. We further quantified the formation of Sc by measuring the FRET signal from bulk solution, as shown in Fig. 3C. The FRET signal of the complete tesseract was ten times higher than that of the small cube alone, indicating that the small cube assembly only took place in presence of all sub-structures of the tesseract. The size of the DNA tesseract was then characterized. The hydrodynamic diameter and diagonal length determined by DLS and Cryo-EM was 25.5 and 19.8 nm, respectively (Fig. 3D). The quality of the measurements was validated by the correlation function and the Cryo-EM density map, respectively (Supplementary Fig. S3). Considering the formation of a double layer on particles during DLS, which results in the hydrodynamic diameter slightly greater than the particle core diameter, the measured sizes by the two techniques were in good agreement [30].

### Stability of DNA tesseract

**Figure 4.**
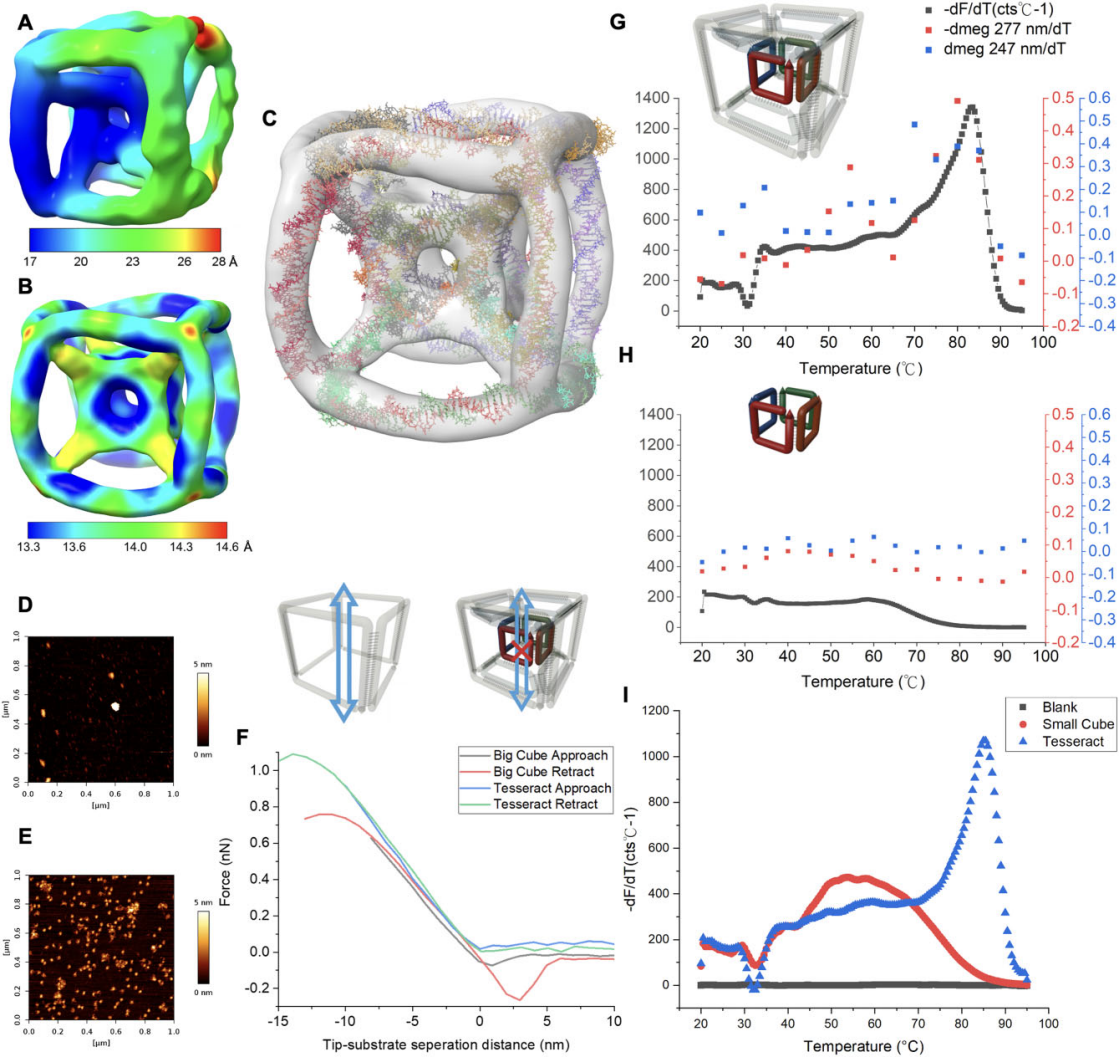
Structure and stability of DNA tesseract. (**A**) Cryo-EM 3D resolution map of the reconstruction of DNA tesseract with C1 symmetry. The global resolution of C1 reconstruction is 21.24 Å. The small cube was found to be more stable than the other parts of the structure. (**B**) The 3D resolution map of reconstruction with octahedral symmetry. The symmetry was applied to enhance the global resolution to 14.16 Å. (**C**) Docking of the simulated model into the density map with octahedral symmetry. The correlation was found to be 78% on chimera. Atomic force microscopy on 20 nM DNA tesseract and the force–distance spectroscopy comparing the (**D**) big cube and (**E**) tesseract. (**F**) Force–distance spectra of big cube and tesseract. The Young’s modulus of the tesseract and big cube were 23.44 ± 0.56 MPa and 19.55 ± 0.98 MPa, respectively. The stiffness of the tesseract as indicated by the energy dissipation (0.033 ± 0.01 × 10^−18^ J) was 23.7 times higher than that of the big cube (0.783 ± 0.1 × 10^−18^ J). 10 random particles were measured (SI Appendix, Supplementary Figs S7 and 8) for the averaged value. (**G**) Thermal stability of tesseract. The melting temperature was determined by melting analysis in quantitative polymerase chain reaction (qPCR) and it was found to be 84°C. The same analysis using CD looking at the signature of B-form DNA showed a similar trend on the melting temperature. (**H**) Thermal stability of small cube. Both qPCR and CD did not show a significant indication of melting temperature due to insufficient amount of DNA. (**I**) Comparison of melting temperature between tesseract and small cube. 1.7 µM of structure was used for enough DNA to generate observable signal. A melting temperature of 54°C was observed from the small cube. The peak disappeared in the tesseract indicating the small cube was stabilized to the same thermal stability.

We next directly examined the tesseract using AFM and Cryo-EM (Fig. 4). We first solved the structure of tesseract by Cryo-EM. The optimization of concentration conditions was essential to avoid aggregation of particles on the Cryo-EM grid (Supplementary Fig. S4). Tesseract-like particles were easily recognized in the raw images (Supplementary Fig. S5A). Three major orthogonal tesseract projections were observed in the 2D classification (Supplementary Fig. S5B). Without applying symmetry, 3D reconstruction (C1) successfully revealed an intact tesseract, in which the Sc was solved at higher resolution compared to the other tesseract components (Fig. 4A). This result suggested that the Sc was the most rigid part of the structure and the hyperstructure might confer a strong mechanical property. In addition to the other stability data above suggesting integrity of the complete tesseract, the fluctuation from the C1 reconstruction was likely caused by damage from freezing in Cryo-EM. Therefore, octahedral symmetry was applied in the reconstruction for a more representative structure as in the bulk solution (Fig. 4B). The simulated model in Fig. 3A was then docked into the octahedral density map using the 3D modeling software package USFC ChimeraX (Fig. 4C), yielding a high-confidence correlation (77.96%) at 14.16 Å between the two structures (Supplementary Fig. S6) [26].

We further confirmed the mechanical property of the tesseract hyperstructure by AFM, revealing evenly distributed particles with the expected two-dimensional size of the tesseract (10 × 10 nm). We measured the force–distance spectrum of the big cube and tesseract with AFM measuring 10 random particles on mica (Fig. 4D and E, 10 replicates in Supplementary Figs S7 and S8). While the Young’s modulus of the tesseract (23.44 ± 0.56 MPa) was similar to that of the big cube (19.55 ± 0.98 MPa), the Young’s modulus was significantly higher than canonical DNA nanostructures previously reported [31–34]. Although *z*-height of the tesseract was distorted when the AFM tip approached, the same modulus observed during retraction, we observed the slope of the force curve as the modulus remained unchanged after the approach and retraction. This showed the fully elastic recovery with excellent structural integrity of DNA tesseract indicating minimal structural hysteresis under deformation. During retraction, the energy dissipation of the complete tesseract (0.033 ± 0.01 × 10^−18^ J) was found to be 23.7 times lower than that of the big cube (0.783 ± 0.1 × 10^−18^ J). This observation suggested that the interior tesseract framework could resist expansion pressure with much higher stiffness, indicating the mechanical strength to survive the acoustic radiation force. Collectively, these data validate the unique ability of DNA tesseract to combine high rigidity with elasticity while maintaining a small size with high density, overcoming a major limitation of conventional DNA architectures for the application under high frequency SAW.

To benchmark the stability of DNA tesseract, we also investigated the melting temperature of the tesseract as the most common characterization of stability of biomolecules. The same combinations of sub-structures were studied. Figure 4G and H, and Supplementary Fig. S9 show the melting of the combinations. Realtime quantitative PCR (qPCR) revealed a positive correlation between melting temperature (*T*_m_) and combination complexity, i.e. number of structure component combinations. The tesseract *T*_m_ was unexpectedly high at 83.5°C to 84°C. To confirm this finding, we also used CD as a function of temperature using 247 nm and 277 nm as the signature peaks of duplex DNA (raw data in Supplementary Fig. S10). CD confirmed tesseract *T*_m_ to exceed 80°C. When the concentration of Sc and tesseract was raised to 1.7 µM, the Sc has enough DNA to generate an observable signal in qPCR showing a melting temperature of 54°C (Fig. 4I). The same peak was not observed from the same concentration of tesseract, indicating the thermal stability of Sc was aligned with the entire tesseract. Finally, to probe stability of the tesseract in a complex environment, we used endonuclease digestion assays whereby DNase-I was employed to determine the structure’s resistance toward degradative stress. In Supplementary Fig. S12, a titration of the tesseract revealed partial degradation starting at 7.7 U/ml DNase, which is about 21 times higher than the physiological levels of DNase-I found in serum (0.36 ± 0.20 U/ml) [35]. We also incubated the DNA tesseract with 95% fetal bovine serum and observed no significant degradation over three days of incubation. These results indicate a marked nuclease resistance of the DNA tesseract and suggests potential applications with clinical sample for biomedical applications.

### Alignment of DNA tesseract into conducting wire

**Figure 5.**
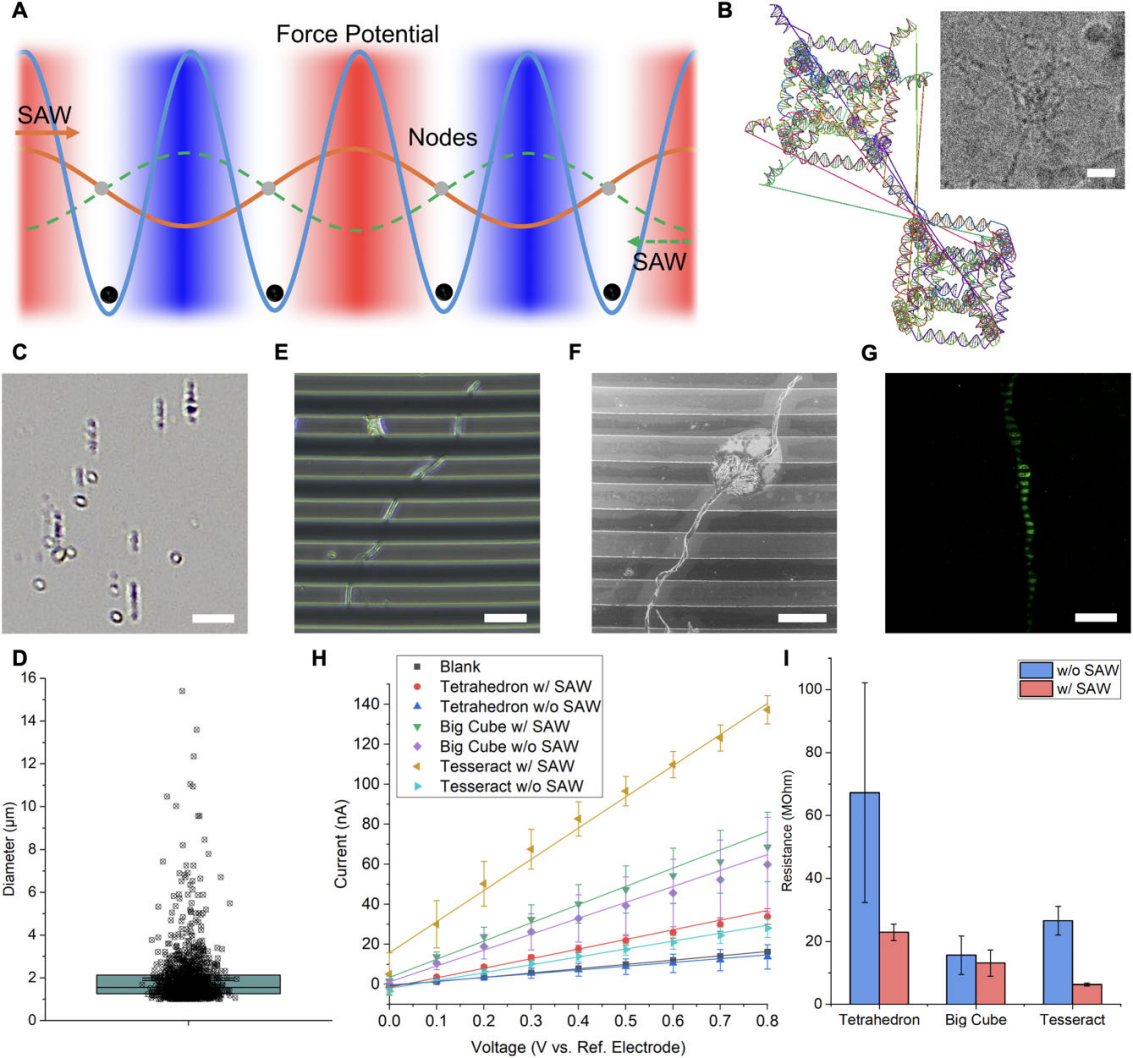
Assembly of DNA tesseract into micro-scale wire for electrical conduction. (**A**) Principle of using SAW to locally concentrate DNA tesseract for clustering. The propagation and reflection of SAW in the capillary would create pressure nodes (gray dots) to further concentrate the DNA tesseracts for the formation of clusters (black dots) as observed in (**B**). (B) Design of tesseract with complementary anchors. Duplexes were added on top of the original design to connect two tesseracts in diagonal manner. Top right: representative Cryo-EM micrograph of single DNA tesseract with overhangs. (**C**) Zoomed-in image from brightfield microscopy observing the presence of DNA tesseract clusters in the glass capillary after application of SAW (342 MHz, 2 V, burst period 30 ms, 1 × 10^6^ cycles, 30 min); scale bar: 10 µm. (**D**) Diameter of 1239 particles was measured with an automated image process implemented in MATLAB. Average diameter was 1.93 µm and median was 1.55 µm. Error bar indicates standard error. (**E**) Representative image of tesseract wire on IDE; scale bar: 20 µm. (**F**) SEM images of the same wire in (E); scale bar: 20 µm. (**G**) Confocal image of a tesseract wire stained with Cy3 anchor; scale bar: 100 µm. (**H**) Measurement of conductivity on IDE. Three different structures before and after the application of SAW were tested. Electrical current readings were recorded at 0.1 V intervals in triplicate and the linear regression was plotted (*R*^2^ ≥ 0.98) for the calculation in (I). Samples were found to be more conductive after the application of SAW. (**I**) Reciprocal of the data in h reflecting the resistance. Samples were found to have lower resistance after the application of SAW.

After characterizing the stabilities of the DNA tesseract, we next investigated the acoustic fabrication of DNA tesseracts into wires to the sub-millimeter scale. Figure 5A illustrates the SAW-driven alignment mechanism: tesseracts accumulate at pressure nodes of the standing wave, enabling accelerated and directional clustering. To allow clustering, eight duplexes were added in Tiamat (Fig. 5B) as overhangs to diagonally connect two tesseracts. The two species of tesseracts—assembled separately and purified via Cryo-EM protocols—were mixed in equimolar ratios, triggering hybridization-driven aggregation (Supplementary Fig. S13A). Then a thin rectangular capillary containing the solution of tesseract clusters was put between two IDTs on a piezoelectric substrate. A standing SAW generated by IDTs was applied across the capillary to align the tesseract aggregate into a stream of large clusters (Fig. 5C) and the optimal time was found to be 30 min (Supplementary Fig. S13B). Prolonged application of SAW would distort the cluster formation. Figure 5D showed the statistics of the diameter of clusters identified from the capillary as average of 1.93 µm and median of 1.55 µm (images for the statistics in Supplementary Fig. S14A). Big cube (BcA, tesseract without trapezoidal prism B, and small cube) and tetrahedron were designed to connect in the same manner as control using the same set of anchors (Supplementary Fig. S14B and C). No clusters were observable in the capillaries after 30 min of SAW. We then pushed the liquid out of the capillary and deposited the liquid on IDE. Thiolated anchors were immobilized onto the electrode for capturing the wire via duplex hybridisation with ssDNA overhangs. Depositing these aligned clusters onto thiol-functionalized IDE yielded continuous wires with sub-millimeter length, as confirmed by brightfield microscopy (Fig. 5E and Supplementary Fig. S15) and scanning electron microscopy (SEM) (Fig. 5F). At least 16 h of overnight incubation was required for the growth of wire on IDE (Supplementary Fig. S16). To further confirm the wire was DNA, a Cy3 anchor was used to stain the electrode, and signal was observed under confocal microscopy (Fig. 5G and Supplementary Fig. S17). In addition, energy-dispersive X-ray spectroscopy in SEM showed the presence of phosphorus and nitrogen as the signature of DNA in the system (Supplementary Fig. S18). To quantitatively measure the effect of wire formation by hypothesizing that a longer wire will have more contacts on IDE, a potentiostat was used to apply a linear voltage across the electrode observing the variation in electric current. In Fig. 5H, it could be observed that the tesseract wire showed higher current (or lower resistance) after the SAW was applied. Structural variants were tested as control using BcA (tesseract without small cube and trapezoidal prism B) and DNA tetrahedron with the same edge length as the tesseract. Apart from the absence of observable wires from these controls, each showed lower current and higher resistance (Fig. 5I) than the completed tesseract wire. These results demonstrate that the mechanical rigidity and thermal stability of our DNA tesseract enable survival under SAW-induced stresses, while its modular overhang design permits scalable, directional assembly. These results indicated the strategy combining SAW and the simplicity of stable DNA tesseract, is a scalable method beyond origami in fabricating large scale DNA based bioelectronics.

## Discussion

In this work, we have demonstrated the use of SAW to produce clusters of DNA nanostructures and the formation of a sub-millimeter wire with observable conductivity. The concept of using SAW to cluster DNA nanostructures was established using DNA origami by Arnon et. al [9]. However, DNA origami has some drawbacks including needing typically >100 staple strands in excess which may preclude translational application. Based on the conceptual foundation built from Arnon *et al.* [9], we determined that for wire formation instead of linear clustering we would need to use an IDT with a higher frequency 342 MHz as compared to 19.34 MHz previously. When assuming identical electromechanical conversion efficiency of IDT across different frequencies, a shorter wavelength of standing wave was introduced into the capillary with smaller peak-to-peak voltage (2 V versus 20 V). As a result, the clusters formed in the standing wave node would be smaller than 2 µm (Fig. 5C and D), such that finer clustering could be achieved. Due to the high frequency of SAW, particles in the capillary would encounter much more significant acoustic turbulence. Therefore, a stable DNA nanostructure with enhanced mechanical strength would be required to survive.

As a solution to the challenge of creating a DNA nanostructure with extreme mechanical strength, we used the programmability of DNA to create a tesseract, by capturing a small cube within a big cube of the same material. Such tesseracts can be fabricated into a conductive wire using SAW. As compared to conventional DNA origamis that typically use a long single strand viral DNA with hundreds of single stranded DNAs, the tesseract is synthesised from only 16 oligonucleotides, yet has a melting temperature ~20°C higher (Fig. 4G–I and Supplementary Fig. S9). The design of DNA tesseract allowed us to put an unstable cargo (Sc) within the structure as observed by Cryo-EM, and stabilized it to the same extent as the entire structure, thermally (Fig. 4I and Supplementary Fig. S9), spatially (Fig. 4A and B), and mechanically (Fig. 4D–F) for survival under acoustic radiation force.

The remarkable stability of the DNA tesseract could provide a structural approach to significantly stabilize DNA for applications in medicine and material sciences with advantages compared to use of chemical modifications [12, 36–38], extreme ionic conditions, or unusual DNA motifs [11, 16, 39, 40]. The tesseract could be useful for the display of biomolecules for structural study [41–43] or for precise molecular scale nano-assembly of catalysts. Sealing the ends of single-stranded DNA on the tesseract by ligation or UV irradiation could further stabilize the structure [44, 45]. The exceptional and unusual properties of the DNA tesseract also suggest potential applications in drug delivery. Functionalizing the tesseract with nucleic acid aptamers for strand displacement and targeted drug delivery [46–49] would further extend the horizons of applications [50]. We anticipate the easily synthesised and accessible DNA tesseract structure, comprising 16 short oligonucleotides prepared by simple thermal assembly, will be beneficial in a wide range of scientific fields.

In addition, we demonstrated the growth of DNA tesseract clusters into a conductive wire. Although the contact between IDT and capillaries could be improved for better transduction of acoustic force, we successfully collected clusters of DNA tesseract for the IDE. There have been a few examples in the literature measuring conductivity of DNA duplexes and DNA origami [6, 8, 51, 52]. The voltage applied in these examples ranged from −0.4 to 10 V obtaining current from −20 to 30 nA. The conductivity of the DNA tesseract wire was high at 130 nA with 0.8 V. The IDE served as a template for the clusters of DNA tesseract coming together as a wire. DNA nanostructures, especially DNA origamis, have previously been used as a template to pattern the growth of organic and inorganic materials [53–58]. With specific design of overhang sequences for tiling, they could also grow into well-defined superstructures at the micron-scale [59–62]. We have also demonstrated coating of gold nanoparticle on the tesseract wire via the same set of anchors. Particles were observed coated on the wire under SEM and the conductivity in terms of current was increased about two times (Supplementary Fig. S19). The simplicity of the 16 oligonucleotides facet-based design of DNA nanostructure coupled to high yield, and the use of SAW for clustering at low concentrations, holds promise for more complex DNA-based electronic circuitry.

## Supplementary Material

gkaf1409_Supplemental_File

## Data Availability

The Cryo-EM density map has been deposited to the EMDB under the accession numbers EMD-67283 and EMD-67284.
