# Supplementary material for: Acoustically seeded fabrication of a DNA tesseract into a conductive wire
Source: Nucleic Acids Res. 2025 Dec 31;53(22):gkaf1409. doi: 10.1093/nar/gkaf1409 (PMC12754780; doi:10.1093/nar/gkaf1409)
Supplement: gkaf1409_Supplemental_File [file gkaf1409_supplemental_file.pdf]

Acoustically Seeded Fabrication of a DNA Tesseract into a Conductive Wire

Supplementary material

Image Analysis and High-Density Particle Characterization

Automated image processing was implemented in MATLAB (R2024b) to quantify high-density particle dimensions from micrographs acquired under brightfield microscope. The analytical workflow was comprised of five stages:

1. Interactive ROI Selection: A graphical interface allowed manual region-of-interest delineation or full-frame analysis of raw TIFF images (16-bit depth, 4000×3000 pixels).
2. Adaptive Preprocessing: Grayscale conversion followed by Gaussian filtering optimized feature preservation while suppressing high-frequency noise.
3. Threshold-Based Segmentation: Intensity thresholding identified particle-containing regions through dark-field morphology detection.
4. Morphometric Quantification: The regionprops algorithm extracted geometric parameters, with scale calibration enabling diameter calculation. Size filtering excluded sub-resolution artifacts.
5. Structured Data Output: Automated generation of annotated micrographs (Supplementary Figure 13 and 14) accompanied by Excel files containing centroid coordinates, equivalent diameters, and experimental metadata.

The code implemented dynamic scale calibration (pixel/μm conversion) and outlier rejection to ensure measurement fidelity. All analyses preserved spatial resolution of original TIFF images (16-bit depth, 2048×2048 pixels).

Image Recognition Code

```
%% Parameter settings for 40μm
scaleBarLength_um = 40;     % Scale bar actual length (in micrometers)
scaleBarPixels = 200;       % Scale bar length in pixels in the image
minParticleSize = 5;        % Minimum particle size (in pixels), used for noise filtering
maxParticleSize = 100;      % Maximum particle size (in pixels)

% Parameter settings for 20μm
% scaleBarLength_um = 20;     % Scale bar actual length (in micrometers)
% scaleBarPixels = 250;       % Scale bar length in pixels in the image
% minParticleSize = 12.5;     % Minimum particle size (in pixels), used for noise filtering
% maxParticleSize = 200;      % Maximum particle size (in pixels)

%% ROI Interactive Selection
% Read the image
originalImg = imread("path_to_image_file.tif");
% Display the image for ROI selection
figure('Name','Please select the analysis region');
imshow(originalImg);
title('Please make a selection:');

% Create a selection dialog
choice = questdlg('Do you want to select a specific analysis region?', ...
    'ROI Selection', ...
    'Manually select ROI','Use entire image','Use entire image');

% Handle user selection
switch choice
    case 'Manually select ROI'
        h = drawrectangle('Color','r');  % Draw a rectangular ROI
        roiPos = round(h.Position);      % Get [x y w h]
        roiImg = imcrop(originalImg, roiPos); % Crop the ROI region
        close;  % Close the temporary image window
```

```
        % Update the processed image to the ROI region
        originalImg = roiImg;
        disp(['Selected ROI region: X=',num2str(roiPos(1)),' Y=',num2str(roiPos(2)),...
            ' Width=',num2str(roiPos(3)),' Height=',num2str(roiPos(4))]);

    case 'Use entire image'
        % Keep the original image unchanged
        close;  % Close the temporary image window
        disp('The entire image will be analyzed.');
    otherwise
        error('User canceled the operation.');
end

%% Image Preprocessing
grayImg = rgb2gray(originalImg);  % Convert to grayscale

% Gaussian filtering for noise reduction (adjust sigma value to control blur)
filteredImg = imgaussfilt(grayImg, 2);

%% Image Segmentation
% Parameter settings (added dark region threshold)
darkThreshold = 70;  % Adjust this value based on the actual image

% Gaussian filtering (adjust sigma value)
filteredImg = imgaussfilt(grayImg, 1.5);  % Reduce blur to retain dark region details

% Dark region detection
bwImg = filteredImg < darkThreshold;

%% Parameter settings for 40μm
scaleBarLength_um = 40;     % Scale bar actual length (in micrometers)
scaleBarPixels = 200;       % Scale bar length in pixels in the image
minParticleSize = 5;        % Minimum particle size (in pixels), used for noise filtering
maxParticleSize = 100;      % Maximum particle size (in pixels)
```

```
% Parameter settings for 20μm
% scaleBarLength_um = 20;     % Scale bar actual length (in micrometers)
% scaleBarPixels = 250;       % Scale bar length in pixels in the image
% minParticleSize = 12.5;     % Minimum particle size (in pixels), used for noise filtering
% maxParticleSize = 200;      % Maximum particle size (in pixels)

%% Calculate Pixel-to-Actual Size Conversion Factor
pixelSize = scaleBarLength_um / scaleBarPixels;  % Micrometers per pixel

%% Region Analysis
stats = regionprops('table', bwImg, 'Area', 'Centroid', 'EquivDiameter');

% Calculate actual diameter and filter out size outliers
diameters_pixel = stats.EquivDiameter;
validIdx = (diameters_pixel >= minParticleSize) & (diameters_pixel <= maxParticleSize);
stats = stats(validIdx, :);
diameters_um = diameters_pixel(validIdx) * pixelSize;

%% Visualize Results
figure;
imshow(originalImg);
hold on;

% Plot detected particles
for i = 1:height(stats)
    center = stats.Centroid(i,:);
    plot(center(1), center(2), 'bo', 'MarkerSize', 8, 'LineWidth',1); % Blue circle
    text(center(1), center(2)+55, num2str(diameters_um(i), '%.1fμm'),... % Text shifted 55 pixels down
        'Color','blue', 'FontSize',6, 'HorizontalAlignment','center');
end

% Add scale bar annotation and custom scale bar (line)
scaleBarLength_px = 250; % Scale bar length in pixels
scaleBarThickness = 1;   % Scale bar line width
scaleBarColor = 'w';     % Scale bar color (white)
```

```
% Calculate scale bar position
xStart = 100; % Line start X coordinate (10 pixels from the left edge)
xEnd = xStart + scaleBarLength_px; % Line end X coordinate
yPos = size(originalImg,1) - 100; % Line Y coordinate (30 pixels from the bottom edge)

% Draw the scale bar line
line([xStart, xEnd], [yPos, yPos],...
    'Color', scaleBarColor, 'LineWidth', scaleBarThickness);

% Add annotation text
text(xStart + scaleBarLength_px/2, yPos - 65,... % Text above the scale bar
    [num2str(scaleBarLength_um) ' μm'],...
    'Color', scaleBarColor, 'FontSize', 8,...
    'HorizontalAlignment', 'center', 'BackgroundColor', 'none');

hold off;

%% Output Statistical Results
fprintf('Total of %d particles detected\n', height(stats));
fprintf('Average diameter: %.2f μm\n', mean(diameters_um));
fprintf('Diameter range: %.2f - %.2f μm\n', min(diameters_um), max(diameters_um));

% Generate data table
particleData = table(...
    (1:numel(diameters_um))', ...  % Particle ID
    diameters_um, ...              % Diameter data
    stats.Centroid(:,1), ...      % X centroid coordinate (pixels)
    stats.Centroid(:,2), ...      % Y centroid coordinate (pixels)
    'VariableNames', {...
        'ParticleID', ...         % Unique particle identifier
        'Diameter_um', ...        % Diameter (micrometers)
        'CentroidX_px', ...       % X coordinate
        'CentroidY_px'});         % Y coordinate
```

```
% Set save path
outputFolder = 'path_to_output_directory';  % Modify to your output directory
if ~exist(outputFolder, 'dir')
    mkdir(outputFolder);  % Automatically create the directory
end

% Generate a timestamped filename
[~, imgName] = fileparts('image_file_name.tif');  % Automatically get the original image filename
timestamp = datestr(now, 'yyyymmdd_HHMMSS');
excelFileName = fullfile(outputFolder, ...
    sprintf('%s_analysis_%s.xlsx', imgName, timestamp));

% Save as Excel file (including metadata)
try
    writetable(particleData, excelFileName, 'Sheet', 'Particle Data');

    % Add metadata
    meta = {
        'Analysis Time', datestr(now, 'yyyy-mm-dd HH:MM:SS')
        'Original Image', 'image_file_name.tif'
        'Scale Bar Length (µm)', scaleBarLength_um
        'Scale Bar Pixels', scaleBarPixels
        'Pixel Size (µm/px)', pixelSize
        'Total Particles', height(particleData)
        };
    writecell(meta, excelFileName, 'Sheet', 'Experiment Parameters');

    fprintf('Data successfully saved to:\n%s\n', excelFileName);
catch ME
    warning('File save failed, error message: %s', ME.message);
end

% Optional: Display a preview of the first 10 rows of data in the command line
disp('Preview of the first 10 particles:');
disp(particleData(1:min(10,height(particleData)), :));
```

Theory of assembly driven by standing SAW

Tesseract clusters assemble into fibers driven by an acoustic force (F^{rad}) provided by SAW field, as described by following equation[1]:

$$F^{rad} = 4\pi\Phi k a^3 E_{ac} sin(2kx) \quad (1)$$

where $k = 2\pi/\lambda$ is acoustic wavenumber, a is the radius of DNA clusters, E_{ac} is acoustic energy density, x is the position of clusters along the direction of SAW propagation, Φ is acoustophoretic contrast factor described by Equation (2):

$$\Phi = \frac{1}{3}\left(\frac{5\rho_{\mathrm{p}} - 2\rho_0}{2\rho_{\mathrm{p}} + \rho_0} - \frac{\beta_{\mathrm{p}}}{\beta_0}\right) \quad (2)$$

where ρ_{p} and ρ_0 are mass densities of DNA clusters and water respectively, β_{p} and β_0 are their respective compressibility. If Φ is positive, DNA aggregates tend to move toward the pressure nodes, otherwise they will be driven to the antinodes.

As DNA aggregates move in water driven by the acoustic radiation force, they will be subject to the friction force from the water named drag force (F^{drag})[2]:

$$F^{drag} = 6\pi\eta a(\langle v_2\rangle - v_p) \quad (3)$$

where η is the dynamic viscosity of water, $\langle v_2\rangle$ is second-order time average velocity from acoustic streaming, and v_p is the velocity of DNA clusters. Acoustic streaming is strongly related to the boundary layer in a confined channel and thermal inhomogeneity in the liquid. In our case, the wavelength is much smaller than sizes of the capillary used for holding DNA on the surface of a SAW devices as well as heat accumulation is strictly limited by using pulse signal input, therefore reducing acoustic streaming dramatically.

The motion of DNA aggregates is determined by the relative magnitude of F^{rad} and F^{drag}. The success of DNA assembly can be achieved when F^{rad} is larger than F^{drag}. Reducing acoustic streaming contributes to a small value of drag force. At the meantime, acoustic radiation force is enhanced by increasing the size of DNA aggregate since $F^{\mathrm{rad}}/F^{\mathrm{drag}}$ is proportional to the square of DNA aggregate size. We clearly observed the clusters of DNA tesseract with the size smaller than the distance between two adjacent nodes standing SAW under an optical microscope and SAM, indicating F^{rad} is dominant enough to assemble DNA aggregates.

Supplementary Figures

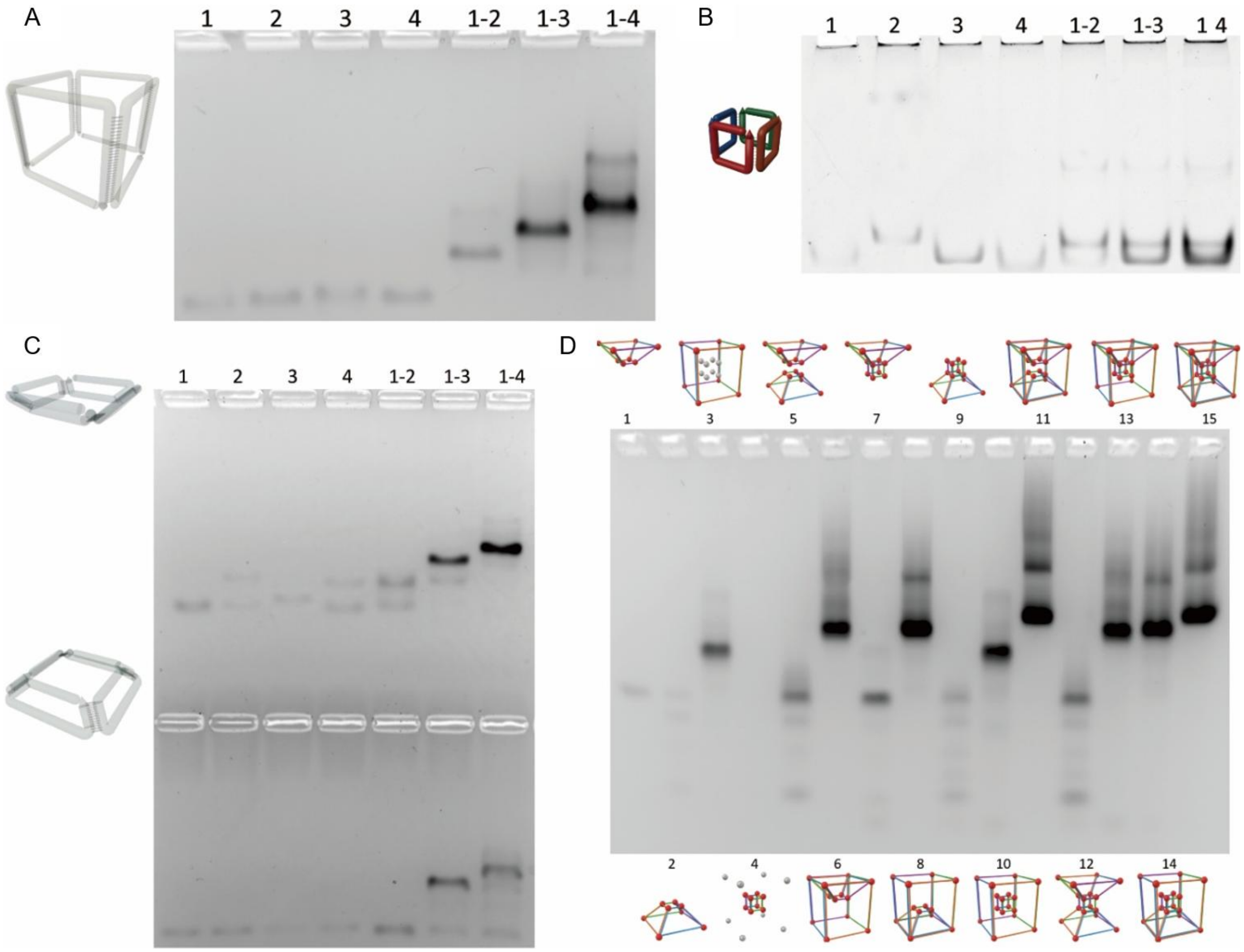

Figure S1. Assembly of individual components and the entire tesseract structure observed by agarose gel electrophoresis. Staining was facilitated by Sybr gold. (**A**) Assembly of large cube. (**B**) Assembly of small cube. (**C**) Assemblies of trapezoidal prism A (upper) and B (lower). Panel **A** and **C** are agarose gel electrophoresis. Panel **B** is polyacrylamide gel electrophoresis. Each component of the DNA tesseract was assembled separately, and the assembly was observed by adding strands from left lane to right lane across the gel. The big cube in **A** and trapezoidal prisms in **c** showed increased molecular size and expected assembly as the number of strands increased. However, the small cube in panel **B** did not assemble alone. (**D**) Partial assemblies of DNA tesseract on agarose gel electrophoresis. Single-stranded DNAs for different combinations of components were mixed for assembly. The small cube was labelled with FRET pairs (two Cy3 and two Cy5). Lane 1: A; Lane 2: B; Lane 3: Bc; Lane 4: Sc; Lane 5: AB; Lane 6: BcA; Lane 7: ScA; Lane 8: BcB; Lane 9: ScB; Lane 10: BcSc; Lane 11: BcAB; Lane 12: ScAB; Lane 13: BcASc; Lane 14: BcBSc; Lane 15: BcABSc (tesseract). By comparing the band intensity of big cube on lane 3 and lane 15, the assembly efficiency of tesseract was found to be 89.5 %.

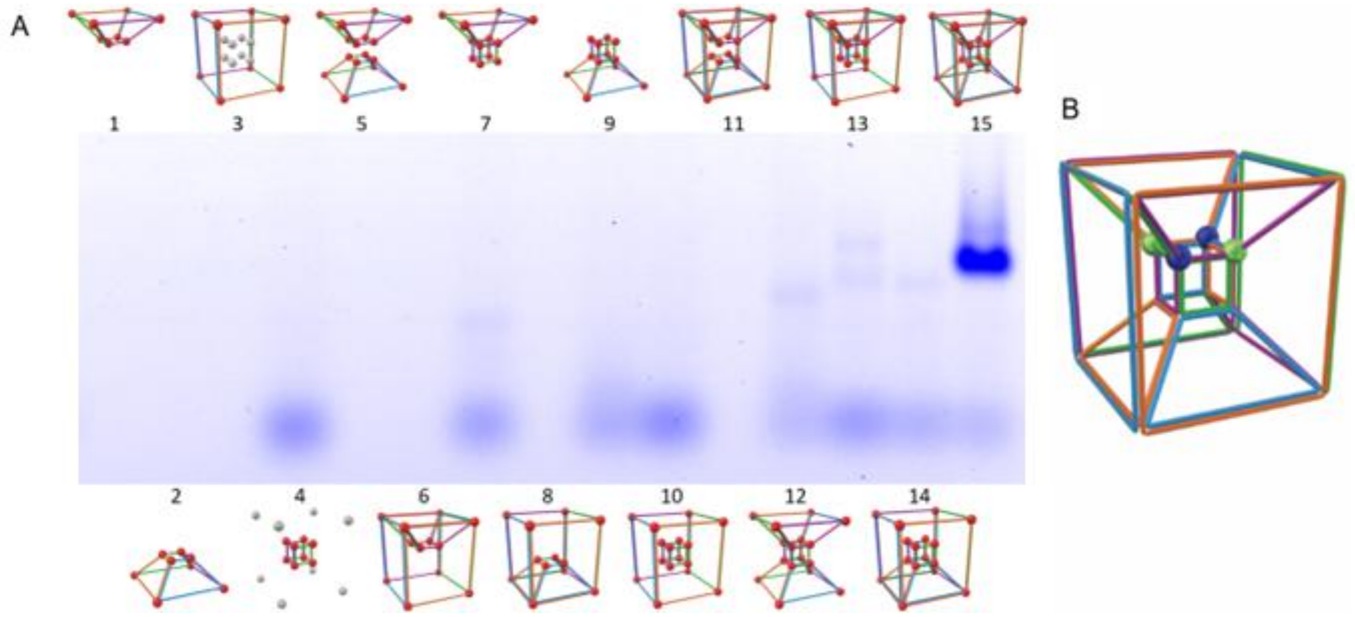

Figure S2. FRET observation of small cube assembly within the DNA tesseract observed by agarose gel electrophoresis. FRET signal was observed on the gel to investigate the small cube structural integrity (**A**) Cy3 and Cy5 FRET pairs were situated diagonally on the small cube (**B**) to allow observation of small cube assembly within the tesseract structure. Blue sphere: Cy3; Green sphere: Cy5. As the FRET signal was not observed for structure 11 but was observed clearly for the full tesseract in structure 15 this indicates stabilization of the small cube within the tesseract. The experiment was performed with 7 hours of electrophoresis. The tesseract FRET intensity remained stable after 7 hours indicating high stability of the tesseract relative to other combinations.

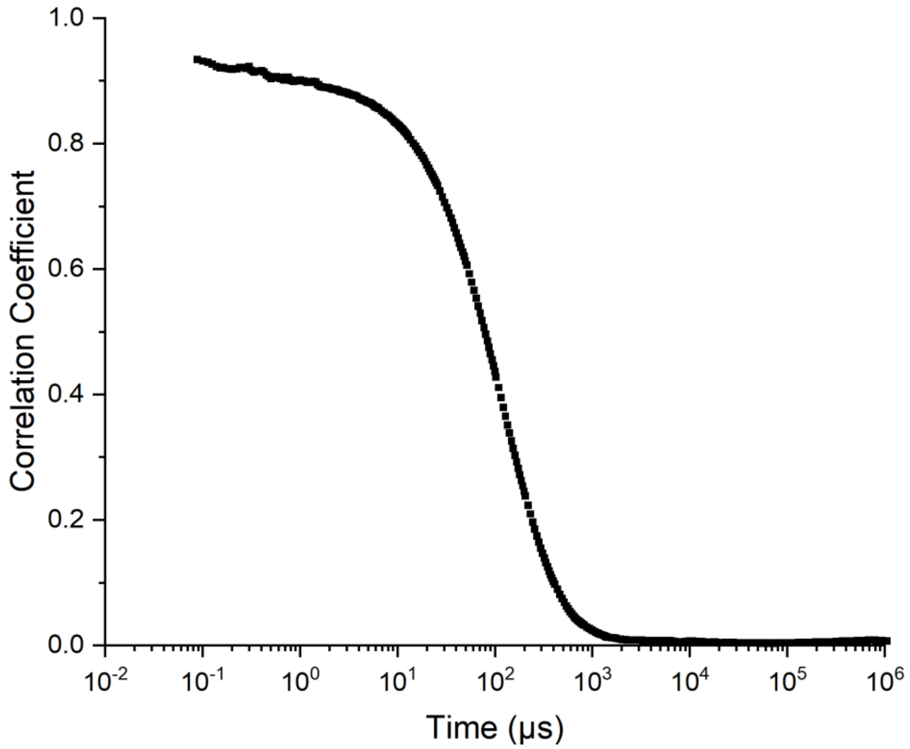

Figure S3. Correlation function of the DLS measurement of DNA tesseract in Figure 3D. The correlation function showed high intercept (> 0.9), smooth and monomodal exponential decay, and a smooth baseline, indicating the high quality of the DLS measurement.

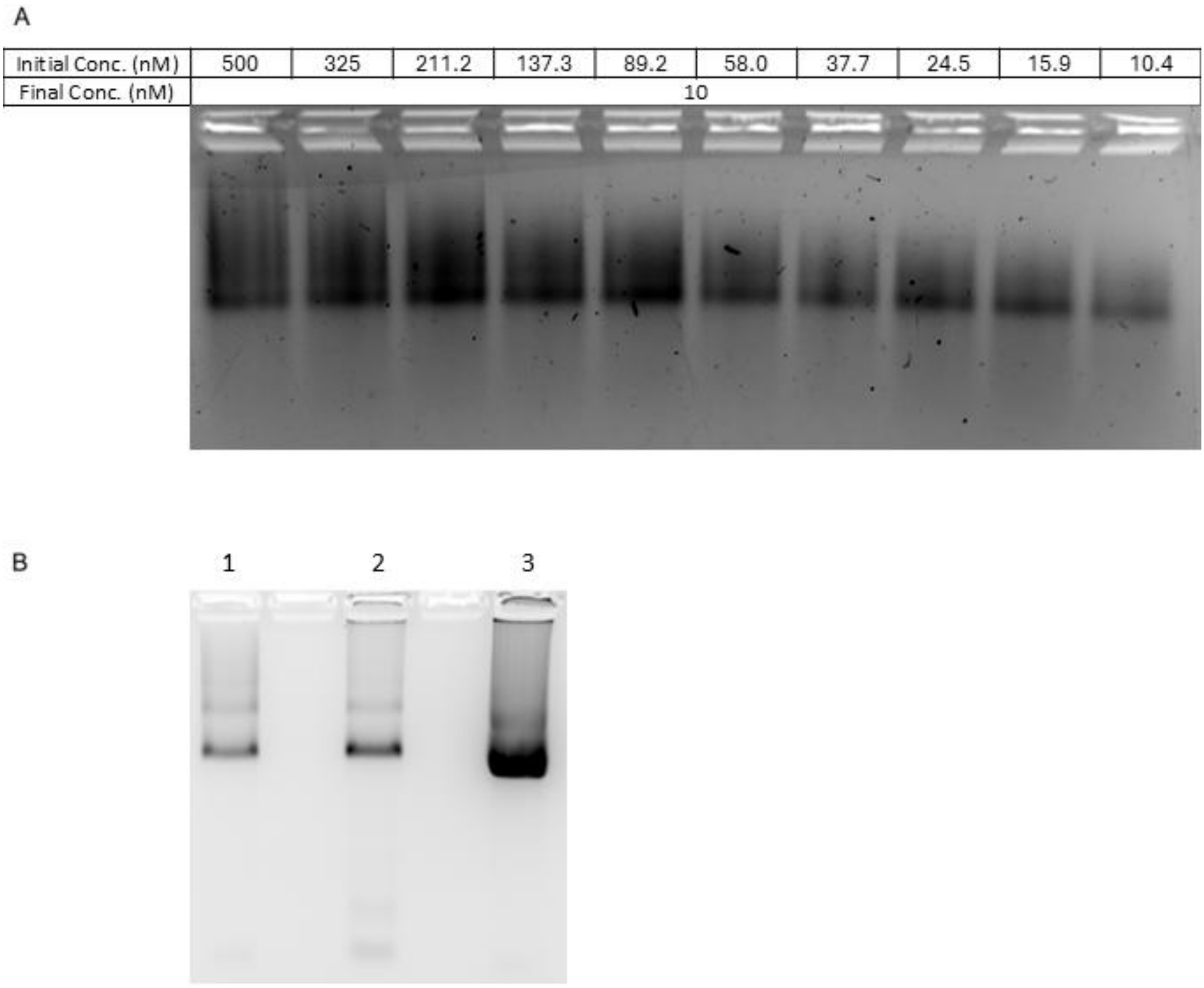

Figure S4. DNA tesseract assembly at different concentrations. (**A**) DNA tesseract was assembled at different concentrations and corrected to the same 10 nM before gel electrophoresis. Significant aggregation of DNA tesseract was observed at concentrations above 37 nM. (**B**) Concentration of 20 nM DNA tesseract for Cryo-EM. 1: Unfiltered 20 nM tesseract, 2: flowthrough in the centrifugal filter, 3: Concentrated tesseract at 10 μM.

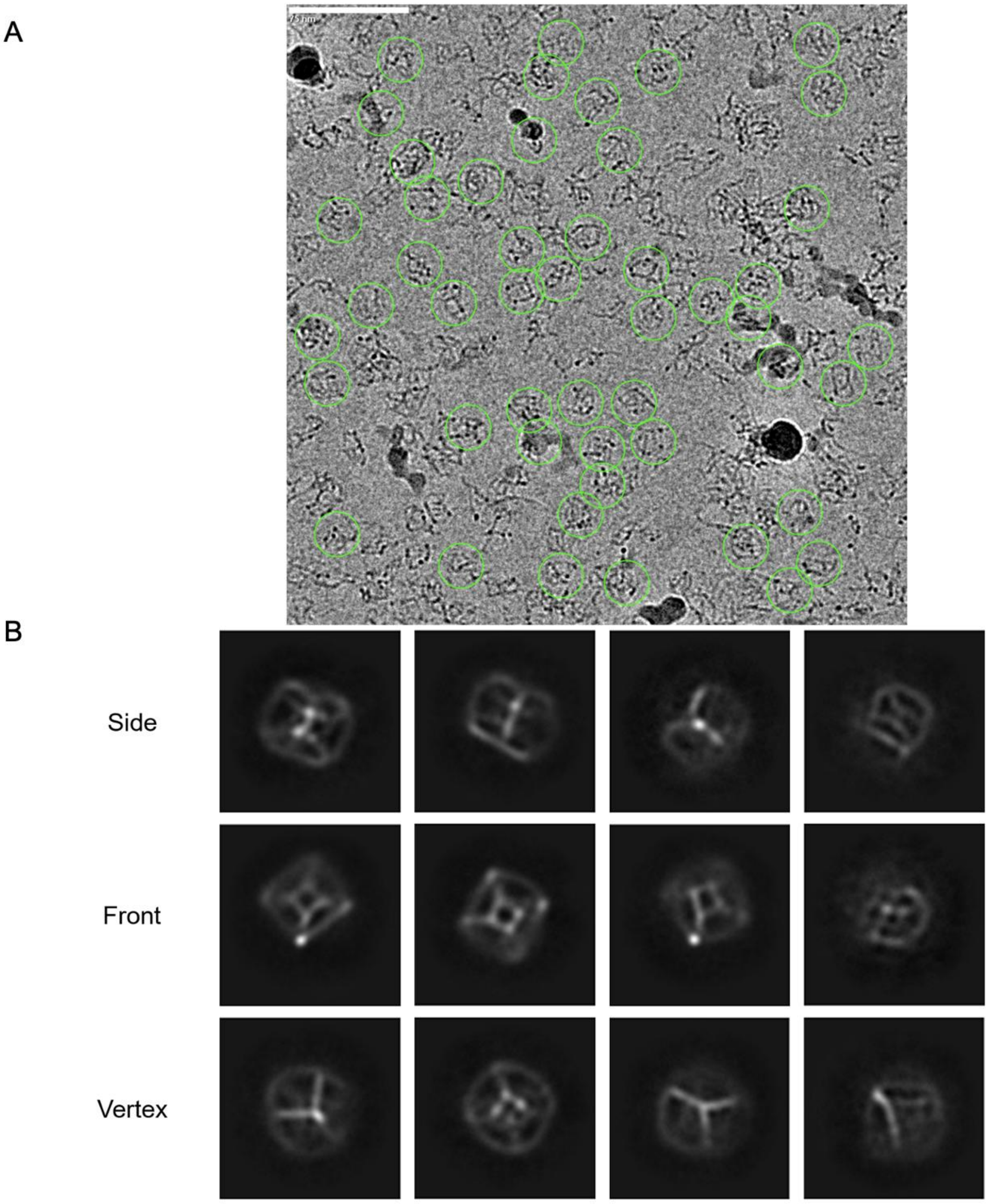

Figure S5. Representative micrograph of Cryo-EM of DNA tesseract and 2D classifications. (**A**) Representative micrograph of topaz picking. Particles that resembled the shape of tesseract were picked with topaz trained model in CryoSPARC. Scale bar in white is 75 nm. (**B**) 2D classifications revealed the three major angles as designed in Figure 2B.

C1 symmetry

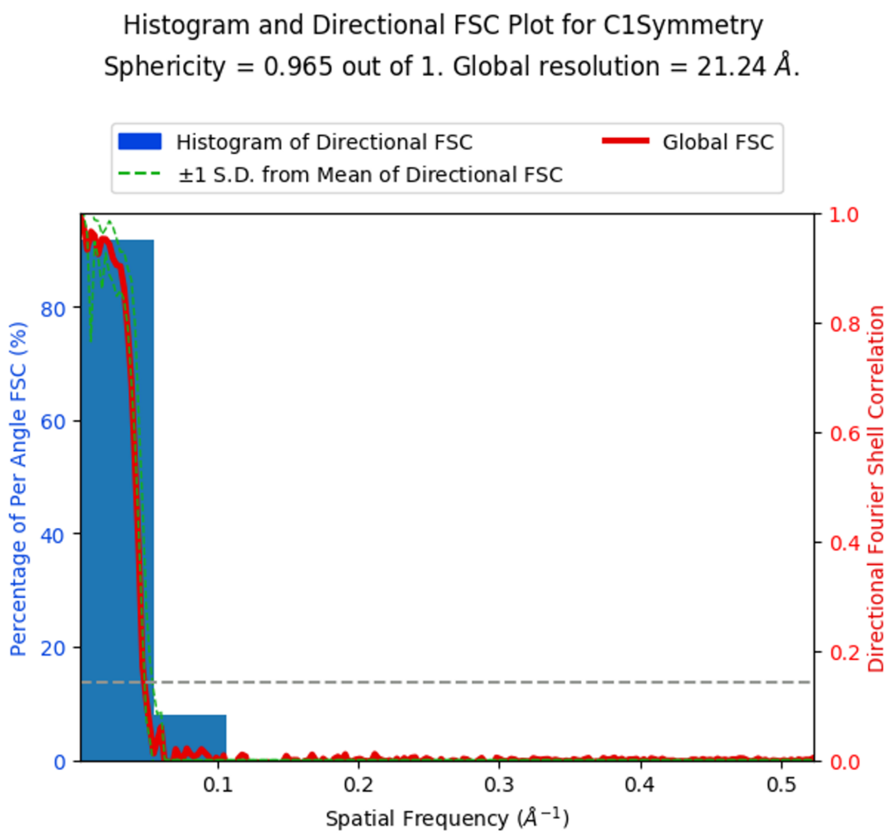

Octahedral symmetry

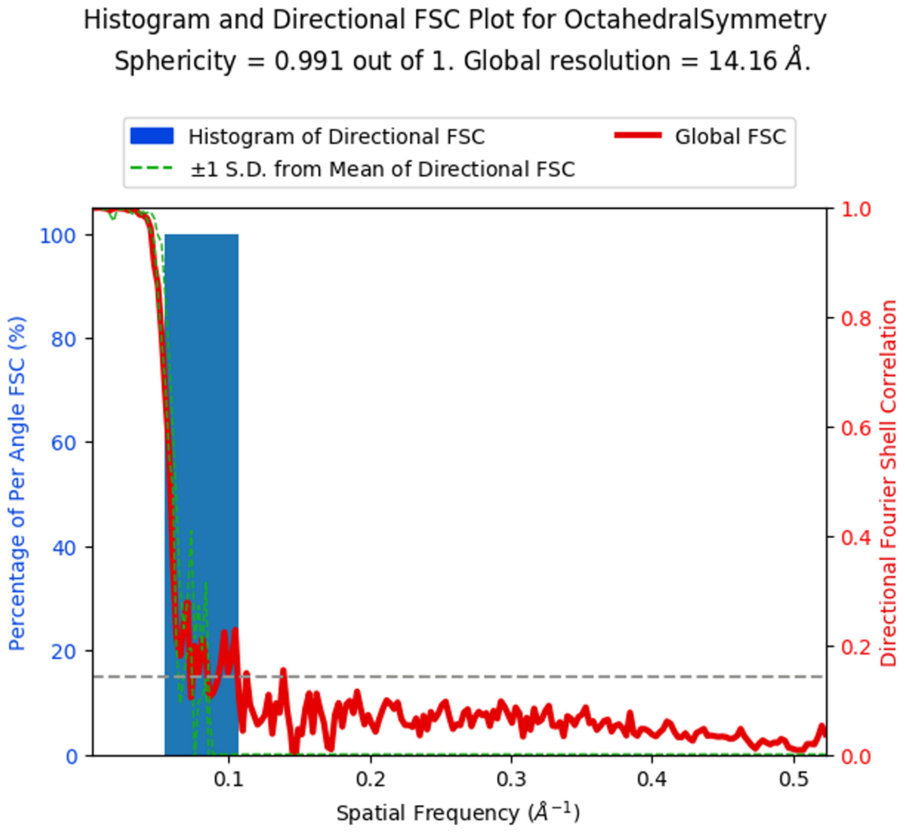

Figure S6. 3D fourier shell correlation determining resolution of reconstruction of DNA tesseract. The resolution determined for C1 and octahedral symmetries were 21.24 Å and 14.16 Å respectively.

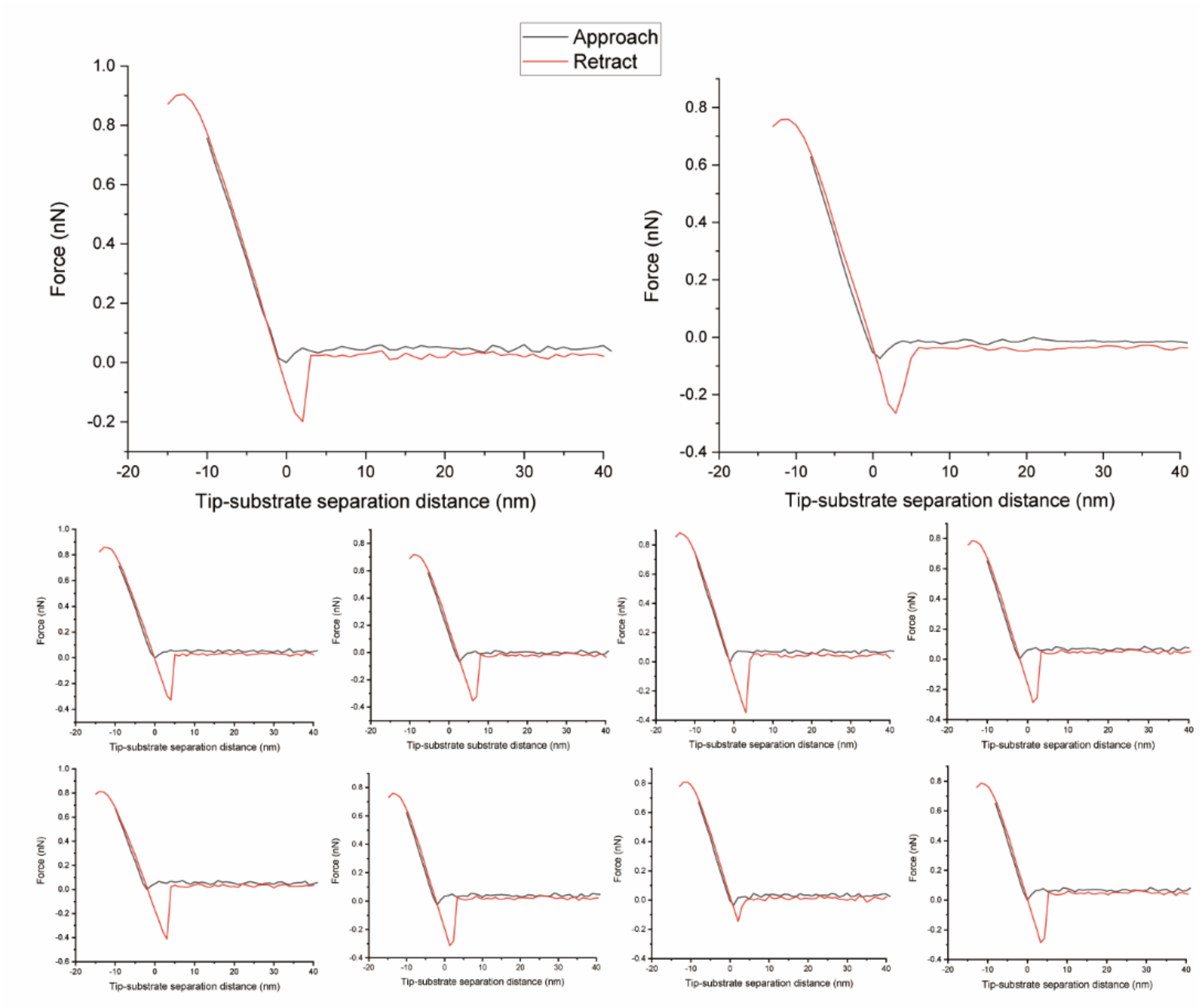

Figure S7. Force-distance spectra of big cube. Individual force-distance spectra of 10 random particles that contributed to the mean spectrum in Figure 4F. Spectra were obtained by approaching the AFM tip towards the particle followed by retraction. Significant adhesion was consistently observed across 10 measurements, indicating the weakness in resisting expansion.

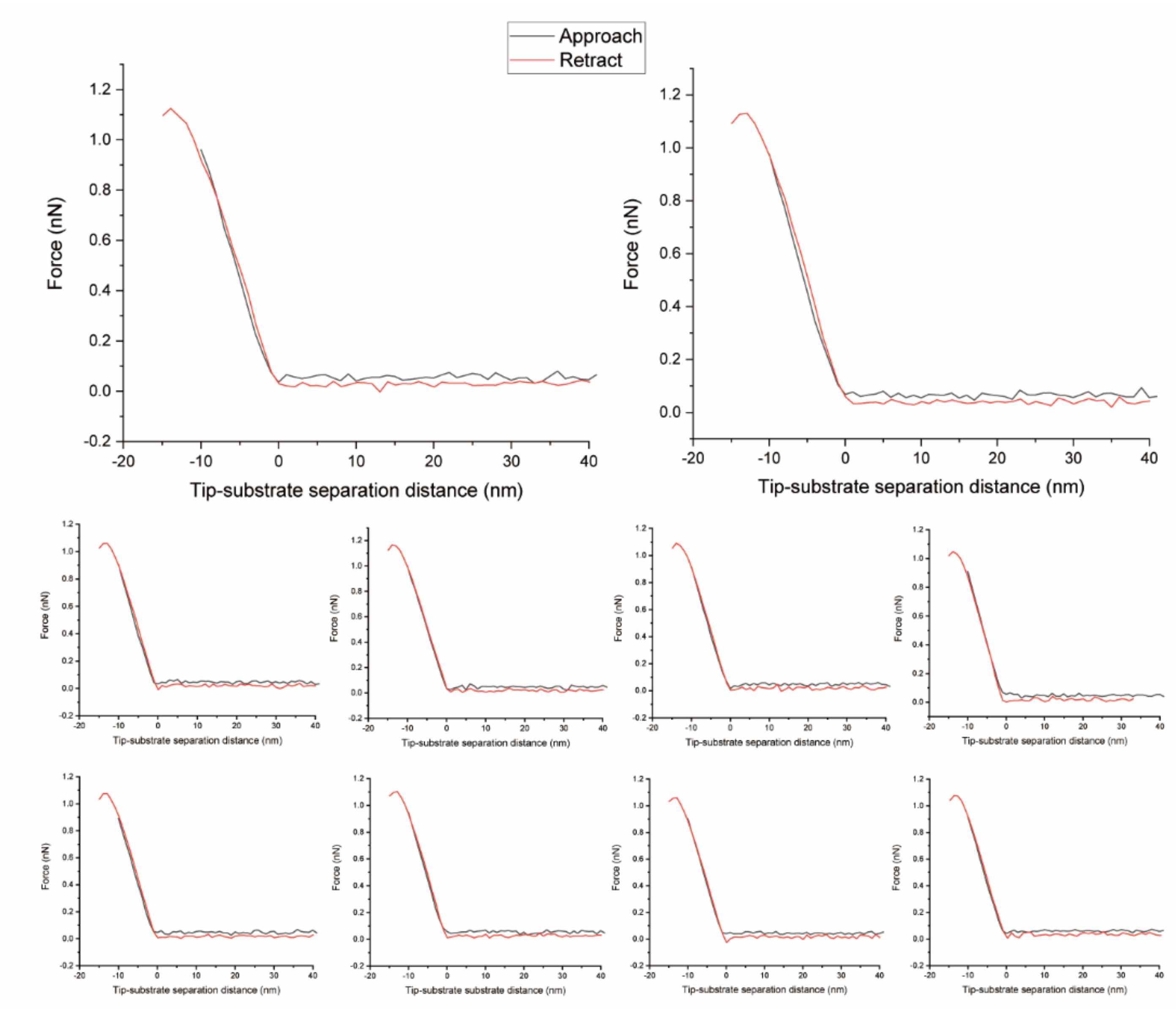

Figure S8. Force-distance spectra of DNA tesseract. Individual force-distance spectra of 10 random particles that contributed to the mean spectrum in Figure 4F. Spectra were obtained by approaching the AFM tip towards the particle followed by retraction. As compared to Figure S7, no adhesion was observed, indicating the comparative strength in resisting expansion.

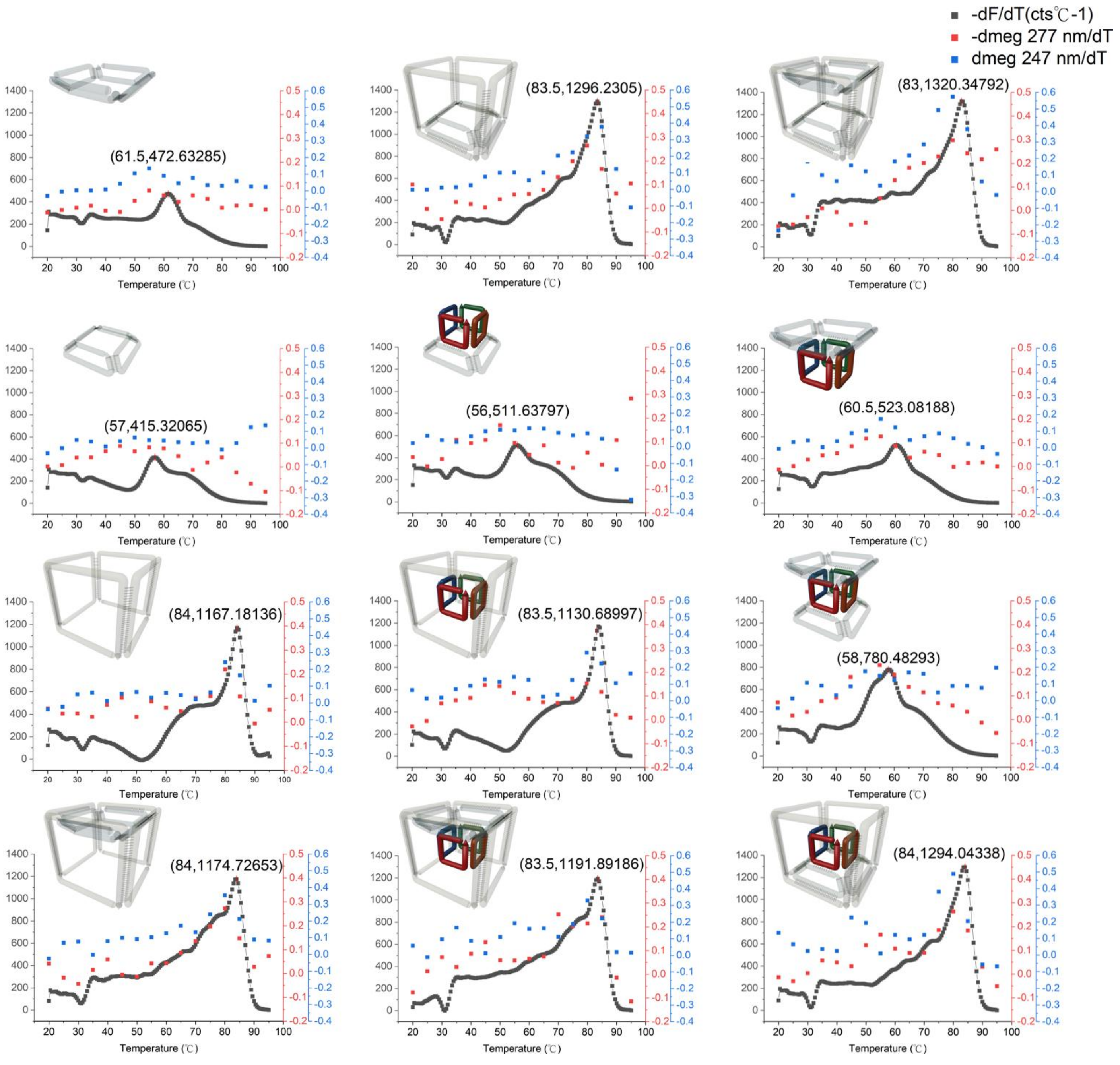

Figure S9. Thermal stability of components of tesseract. The combinations studied on gel electrophoresis were tested by melting analysis in quantitative polymerase chain reaction (qPCR) equipment and circular dichroism (CD) to examine the melting temperature. In qPCR, the signal of Sybr green was measured to detect the integrity of the duplex during melting. In CD, spectra were scanned from 220 nm to 320 nm. Derivatives from the peak value at 247 nm and 277 nm on Figure SS11 were calculated and overlaid with the qPCR data to determine the melting temperature. It could be observed that combinations with the big cube have melting temperatures around 83.5 to 84 °C.

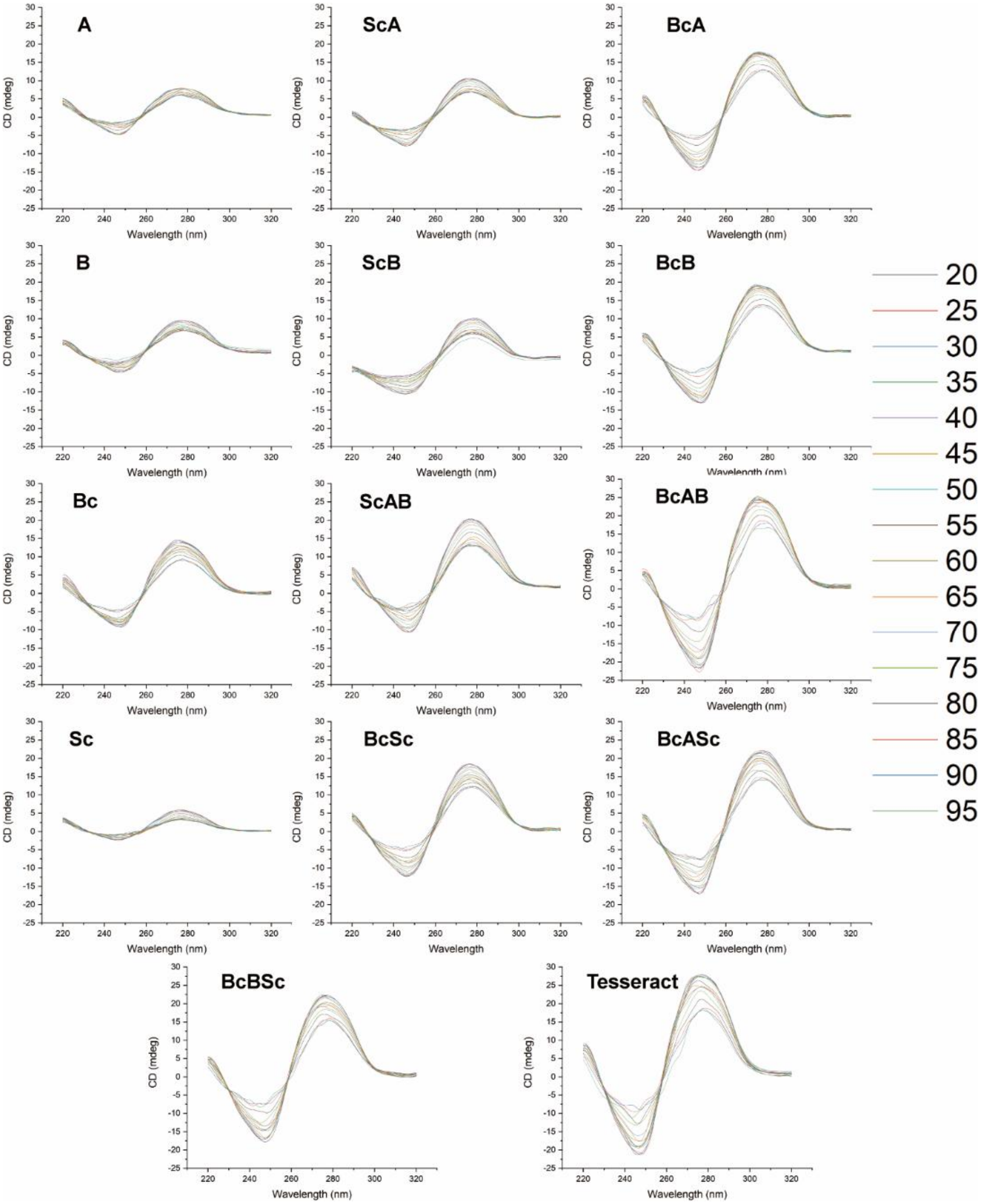

Figure S10. Observation of DNA melting of tesseract and tesseract components by circular dichroism. CD spectra were scanned from 220 nm to 320 nm with an increase in temperature from 20 °C to 95 °C. The signature of DNA duplex was observed with a negative peak at 247 nm and a positive peak at 277 nm. As temperature increased, the CD signal would reduce.

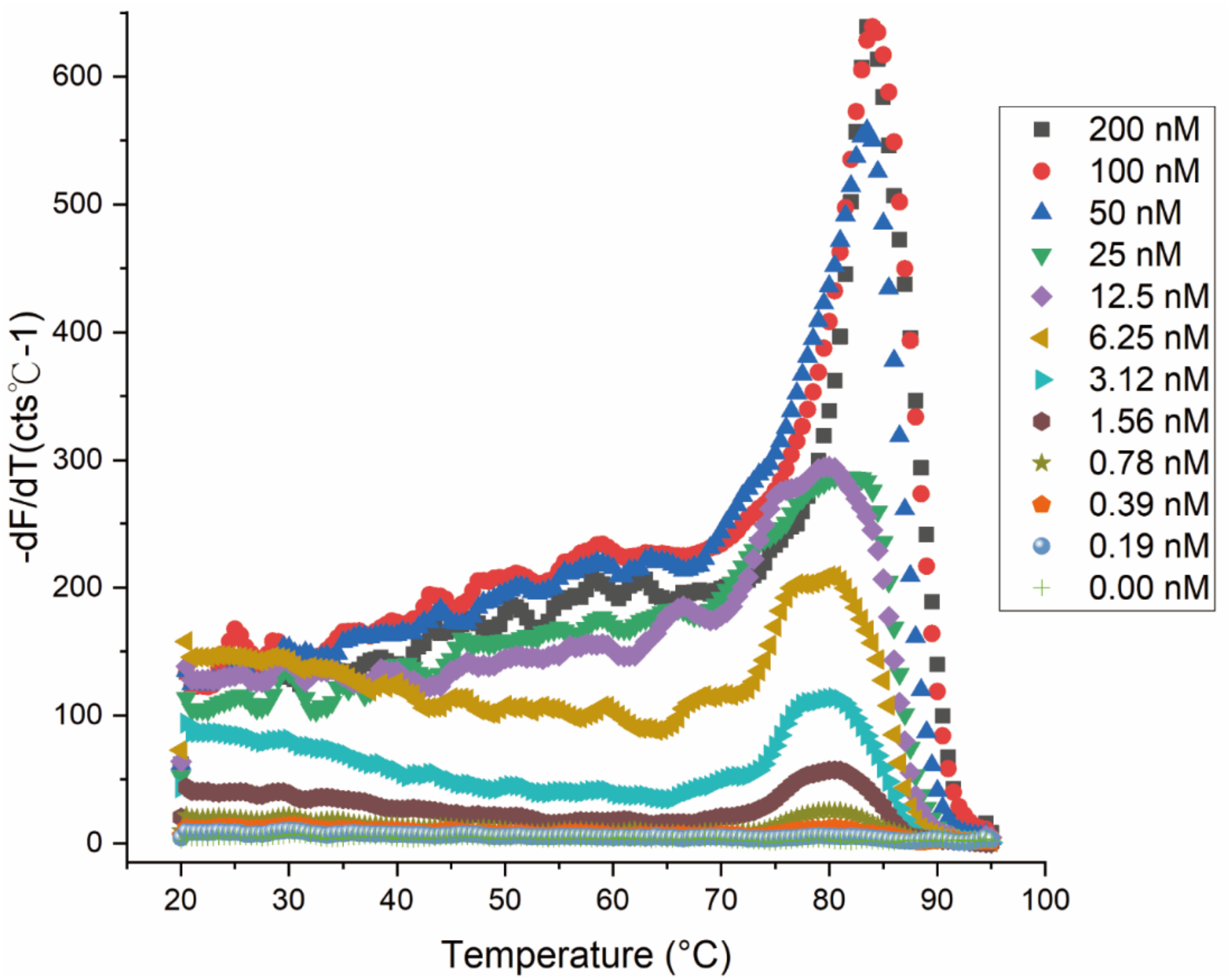

Figure S11. qPCR melting of DNA tesseract at different concentrations. Distinguishable peak value was observable starting from 50 nM onwards.

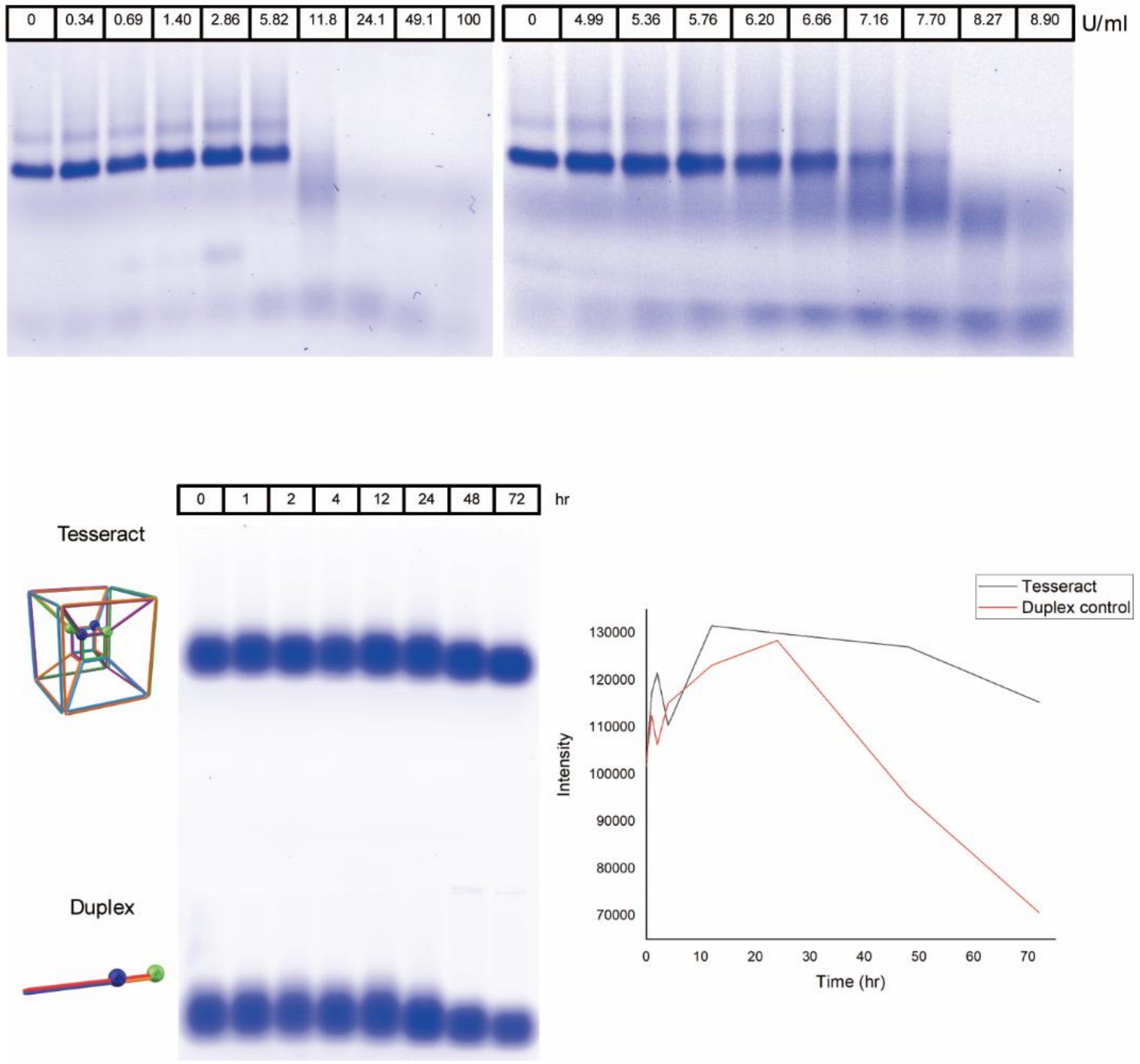

Figure S12. Nuclease resistance of the DNA tesseract observed by FRET signal on agarose gel electrophoresis. Top. Titration of 15 nM DNA tesseract with increasing concentrations of DNase I with a wide range of concentrations (left) and a narrow range of concentrations (right). Degradation of DNA tesseract was observed around 7.70 U/ml. Bottom. Incubation of 15 nM DNA tesseract and a duplex (same distance between the FRET pair) in 95% FBS. Degradation of duplex occurs after 24 hours while the DNA tesseract remained stable for three days. Blue sphere: Cy3; Green sphere: Cy5.

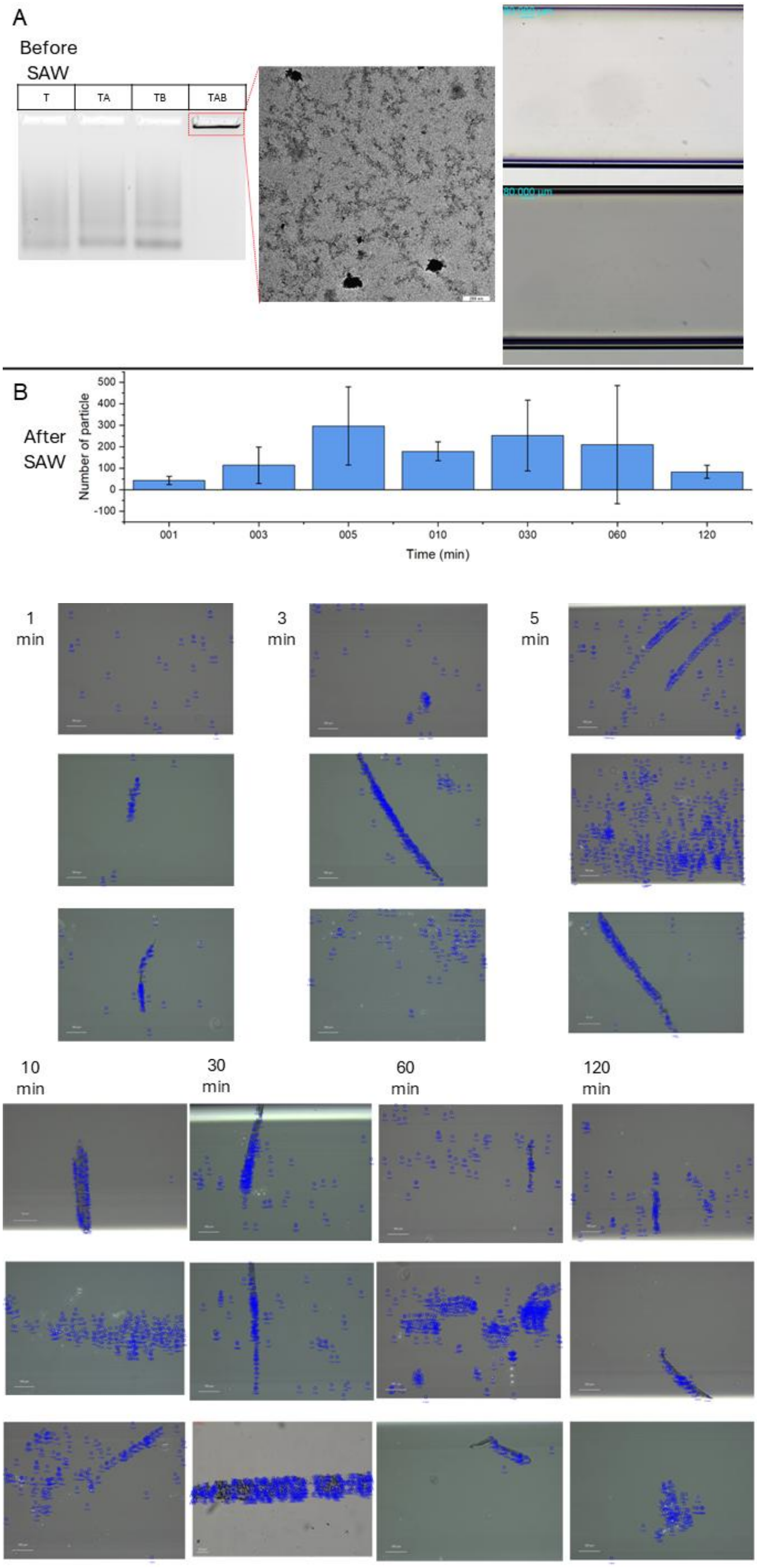

Figure S13. Representative images from brightfield microscope observing the formation of aggregates upon the application of SAW. (**A**) Formation of tesseract aggregate. Tesseract with two set of overhangs were mixed and large aggregate was observed from gel electrophoresis and TEM but not under brightfield microscope with low magnification before application of SAW. (**B**) Aligned particles were observed in the capillary after the application of SAW.

A

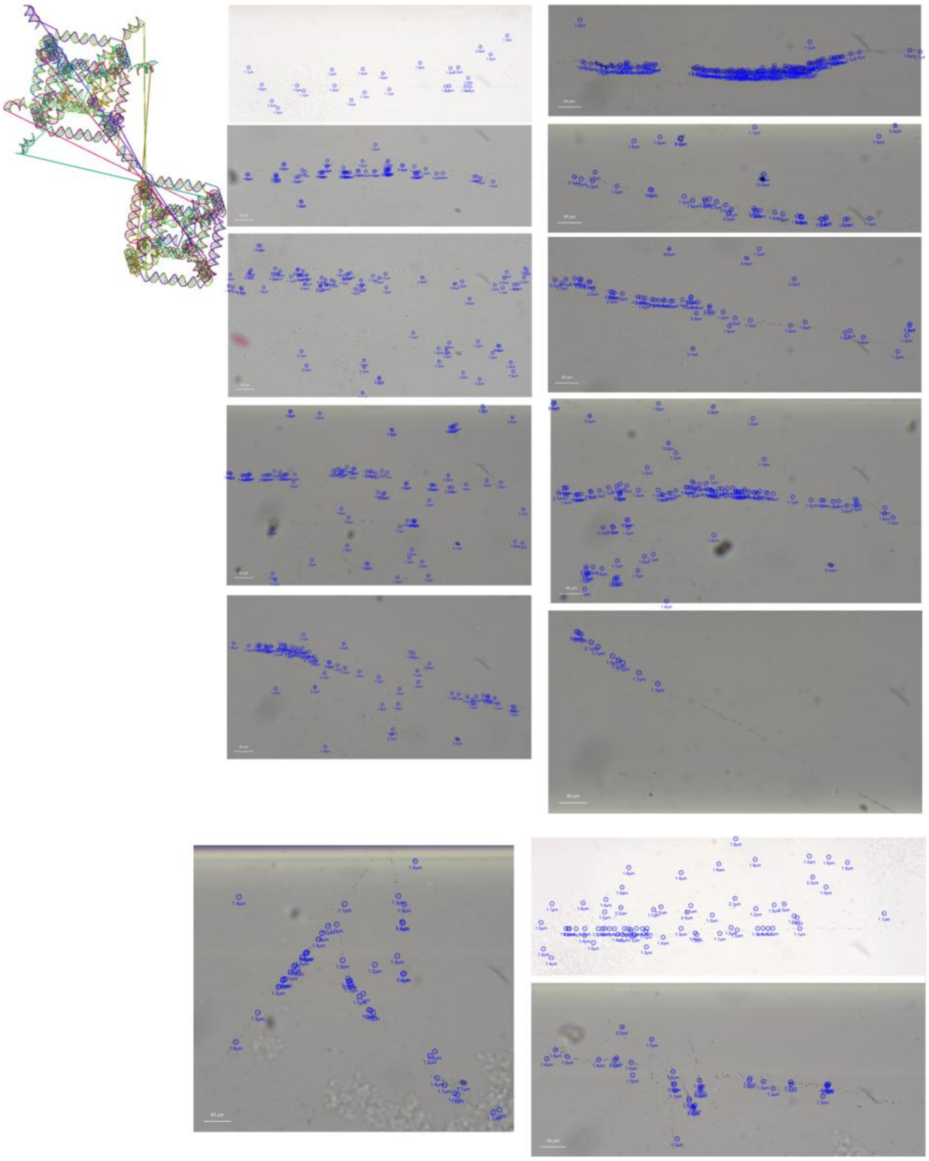

B

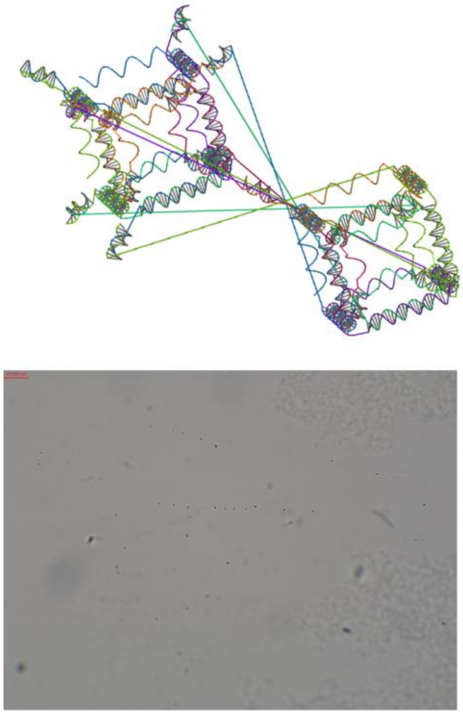

C

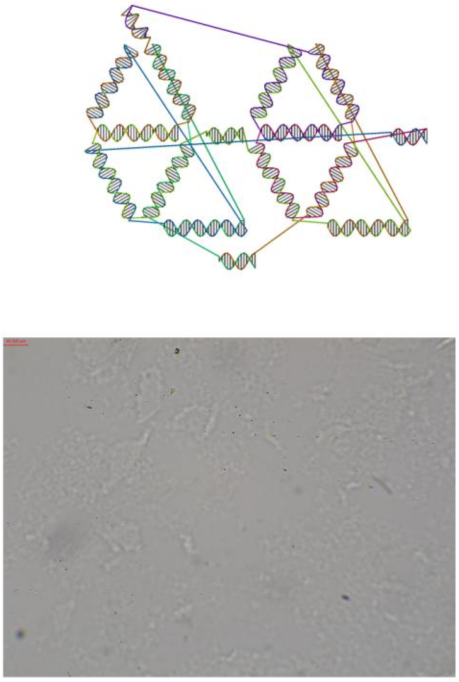

Figure S14. Images from brightfield microscope observing the formation of cluster upon 30 minutes application of SAW. (**A**) Aligned clusters of DNA tesseracts in the capillary were picked up by the automated image processing for the statistics of cluster size in Fig. 5D. (**B**) Design of big cube (BcA, tesseract without trapezoidal prism B and small cube) with the same complementary anchors. Duplexes were added to connect two units in diagonal manner. No clusters were observable in the capillary after 30 minutes of surface acoustic wave. (**C**) Design of tetrahedron with four pairs of the same complementary anchors. Duplexes were added to connect two units alternatively. No clusters were observable in the capillary after 30 minutes of surface acoustic wave.

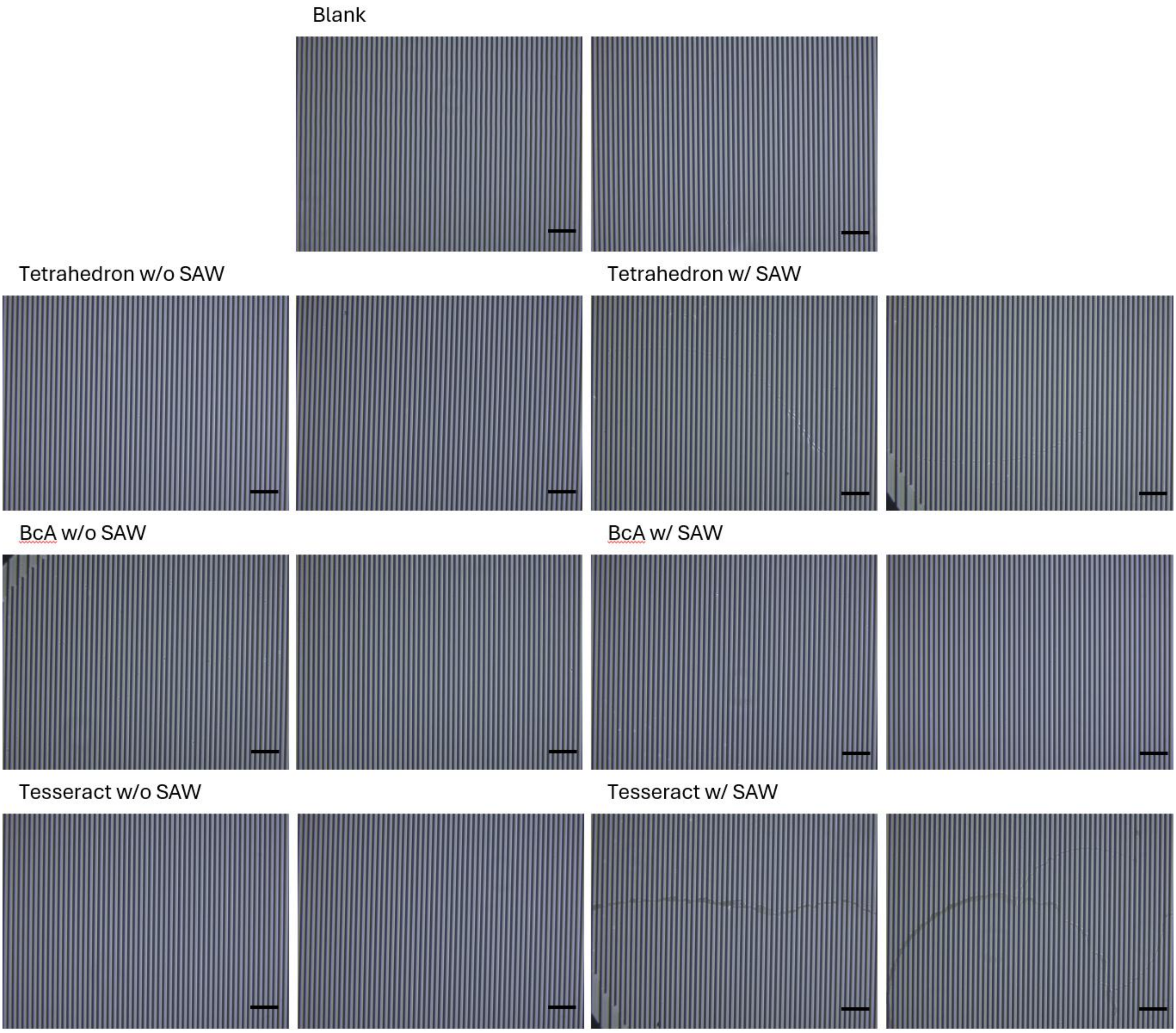

Figure S15. Representative brightfield microscopy micrographs of IDE. The electrodes were deposited with PBS, tetrahedron, BcA or tesseract. Wire was only observed for samples with SAW applied. Scale bar: 100 μm.

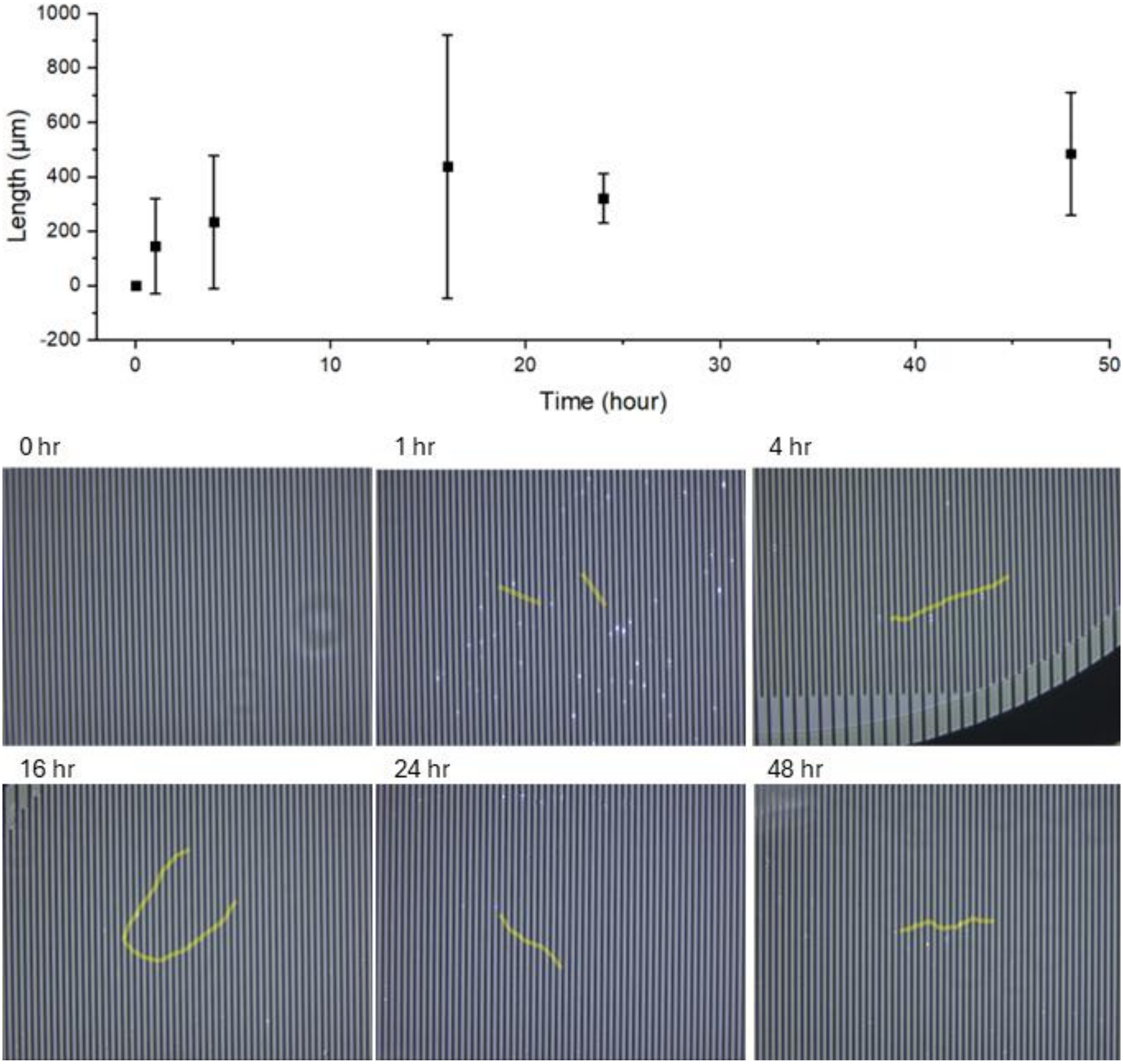

Figure S16. Growth of tesseract wire on IDE along time. The cluster from capillaries were incubated on IDE for various period of time and the lengths observed were measured by ImageJ. Representative images of each incubation time were shown.

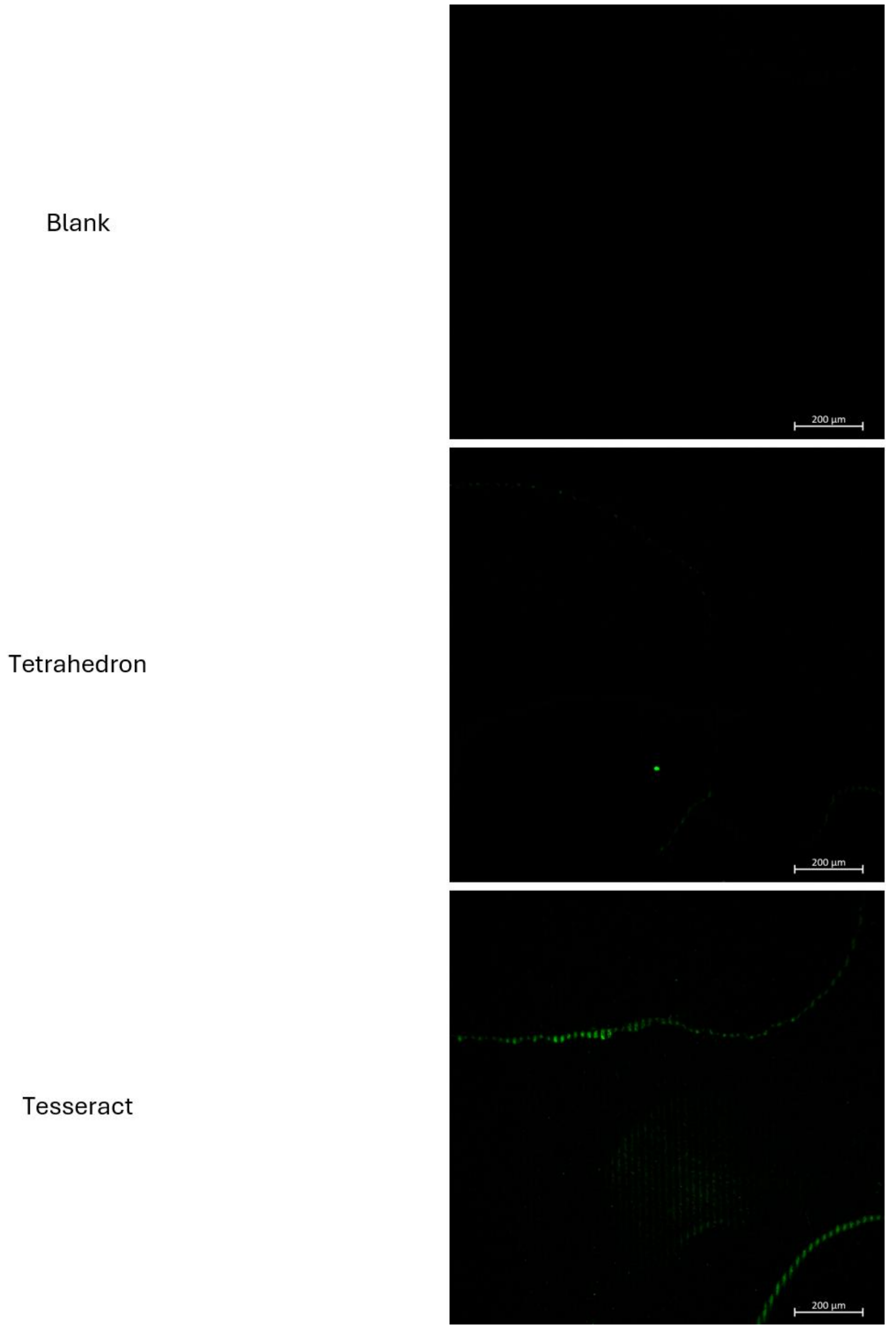

Figure S17. Confocal microscopy images of IDE. IDEs with observable wire from Figure S16 were stained with 10 µl of 1 µM Cy3 anchor for imaging. Cy3 signal was observed where a wire could be identified under brightfield microscope.

IDE between electrodes

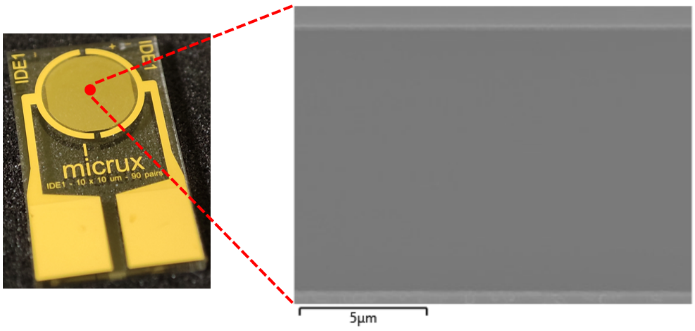

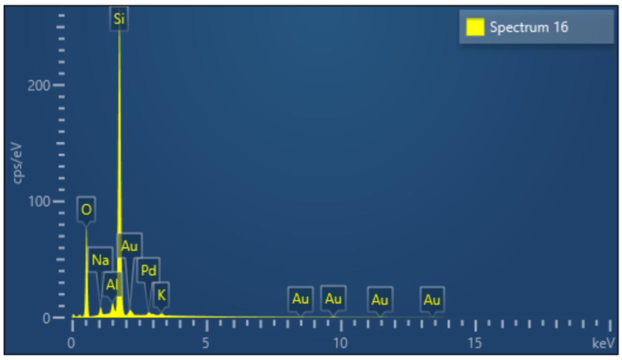

Quant Results:

Element	Line Type	Apparent Concentration	k Ratio	Wt%	Wt% Sigma	Standard Label	Factory Standard
O	K series	103.45	0.34813	45.79	0.12	SiO2	Yes
Na	K series	4.80	0.02026	1.94	0.03	Albite	Yes
Al	K series	3.76	0.02699	1.50	0.02	Al2O3	Yes
Si	K series	116.73	0.92501	44.34	0.11	SiO2	Yes
K	K series	1.46	0.01236	0.60	0.02	KBr	Yes
Pd	L series	3.66	0.03661	1.99	0.06	Pd	Yes
Au	M series	5.95	0.05954	3.84	0.10	Au	Yes
Total:				100.00			

Quant Results:

Element	Line Type	Apparent Concentration	k Ratio	Wt%	Wt% Sigma	Standard Label	Factory Standard
C	K series	1.61	0.01611	100.00	0.00	C Vit	Yes
N	K series	0.00	0.00000	0.00	103.07	BN	Yes
P	K series	0.00	0.00000	0.00	2.63	GaP	Yes
S	K series	0.00	0.00000	0.00	2.38	FeS2	Yes
Total:				100.00			

IDE electrode

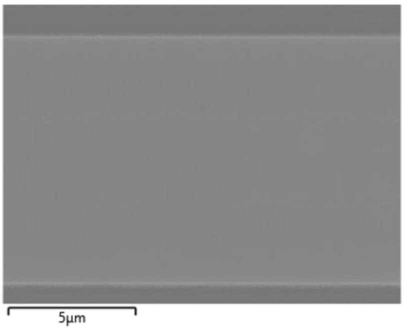

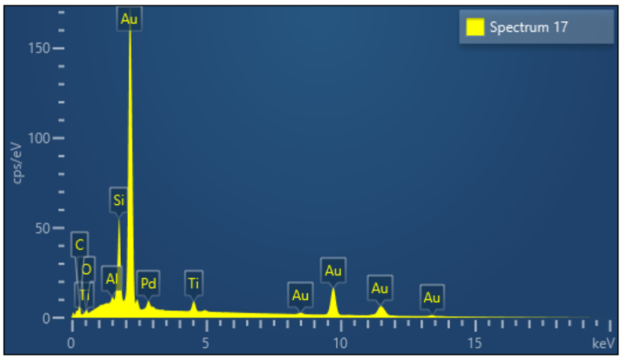

Quant Results:

Element	Line Type	Apparent Concentration	k Ratio	Wt%	Wt% Sigma	Standard Label	Factory Standard
C	K series	5.84	0.05841	8.85	0.45	C Vit	Yes
O	K series	3.19	0.01075	1.94	0.09	SiO2	Yes
Al	K series	1.51	0.01086	0.53	0.02	Al2O3	Yes
Si	K series	19.33	0.15314	5.77	0.05	SiO2	Yes
Ti	K series	6.21	0.06213	2.45	0.04	Ti	Yes
Pd	L series	5.19	0.05194	3.05	0.09	Pd	Yes
Au	M series	219.14	2.19136	77.42	0.40	Au	Yes
Total:				100.00			

Quant Results:

Element	Line Type	Apparent Concentration	k Ratio	Wt%	Wt% Sigma	Standard Label	Factory Standard
C	K series	4.32	0.04324	57.71	5.78	C Vit	Yes
N	K series	1.91	0.00339	42.29	5.78	BN	Yes
P	K series	0.00	0.00000	0.00	1.32	GaP	Yes
S	K series	0.00	0.00000	0.00	1.10	FeS2	Yes
Total:				100.00			

Tesseract wire

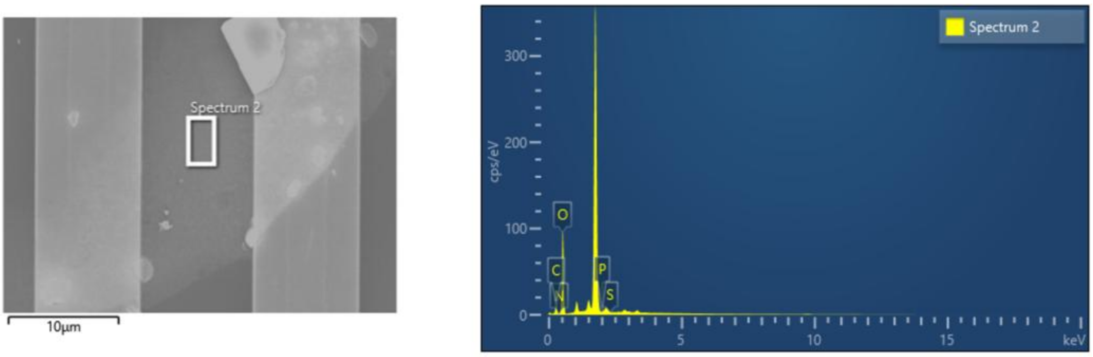

Quant Results:

Element	Line Type	Apparent Concentration	k Ratio	Wt%	Wt% Sigma	Atomic %	Standard Label	Factory Standard
C	K series	10.11	0.10112	19.83	0.22	24.83	C Vit	Yes
N	K series	0.15	0.00027	0.10	0.32	0.10	BN	Yes
O	K series	128.69	0.43305	79.54	0.33	74.80	SiO2	Yes
P	K series	0.90	0.00505	0.54	0.05	0.26	GaP	Yes
S	K series	0.00	0.00000	0.00	0.04	0.00	FeS2	Yes
Total:				100.00		100.00		

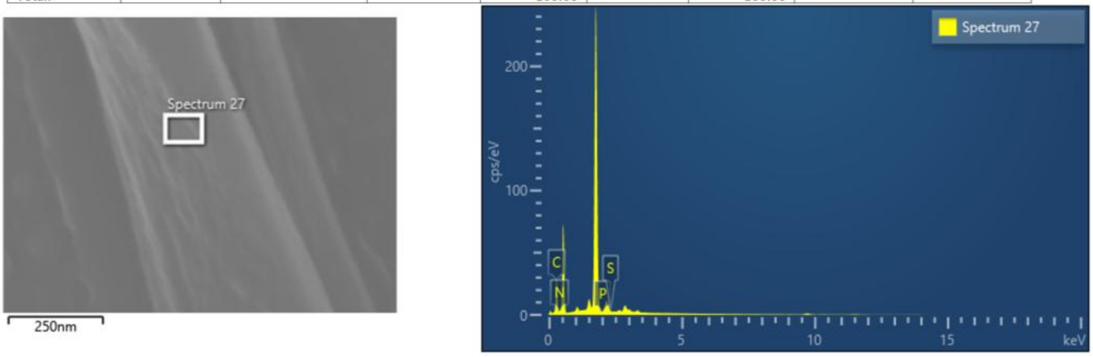

Quant Results:

Element	Line Type	Apparent Concentration	k Ratio	Wt%	Wt% Sigma	Standard Label	Factory Standard
C	K series	24.54	0.24536	58.79	1.58	C Vit	Yes
N	K series	10.08	0.01795	41.21	1.58	BN	Yes
P	K series	0.00	0.00000	0.00	0.10	GaP	Yes
S	K series	0.00	0.00000	0.00	0.08	FeS2	Yes
Total:				100.00			

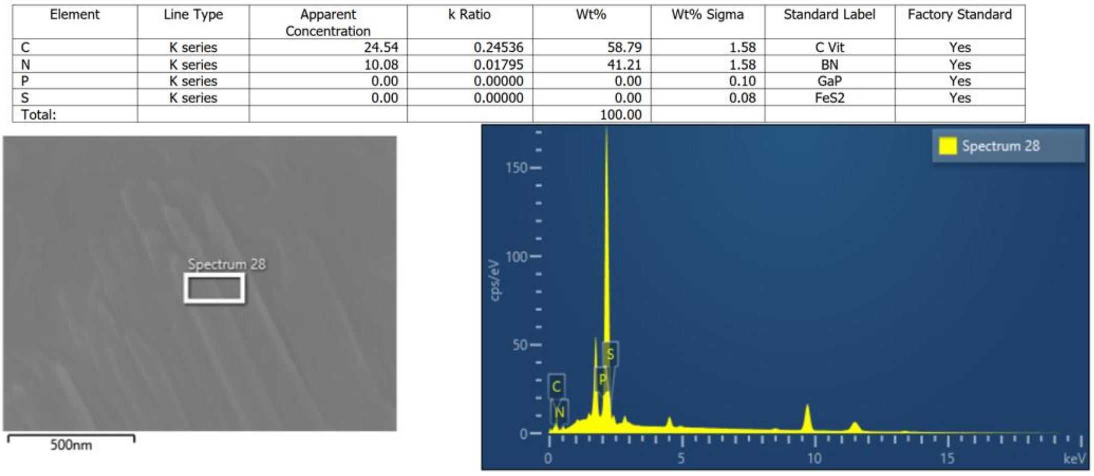

Quant Results:

Element	Line Type	Apparent Concentration	k Ratio	Wt%	Wt% Sigma	Standard Label	Factory Standard
C	K series	9.33	0.09333	55.86	2.41	C Vit	Yes
N	K series	4.65	0.00827	44.14	2.41	BN	Yes
P	K series	0.00	0.00000	0.00	0.59	GaP	Yes
S	K series	0.00	0.00000	0.00	0.49	FeS2	Yes
Total:				100.00			

Figure S18. Energy dispersive X-ray spectroscopy in SEM. Elements on the IDE was mapped to identify the presence of DNA. Blank was mapped to observe the presence of background elements. Trace amount nitrogen and phosphorus were only observed in the region where a wire could be identified.

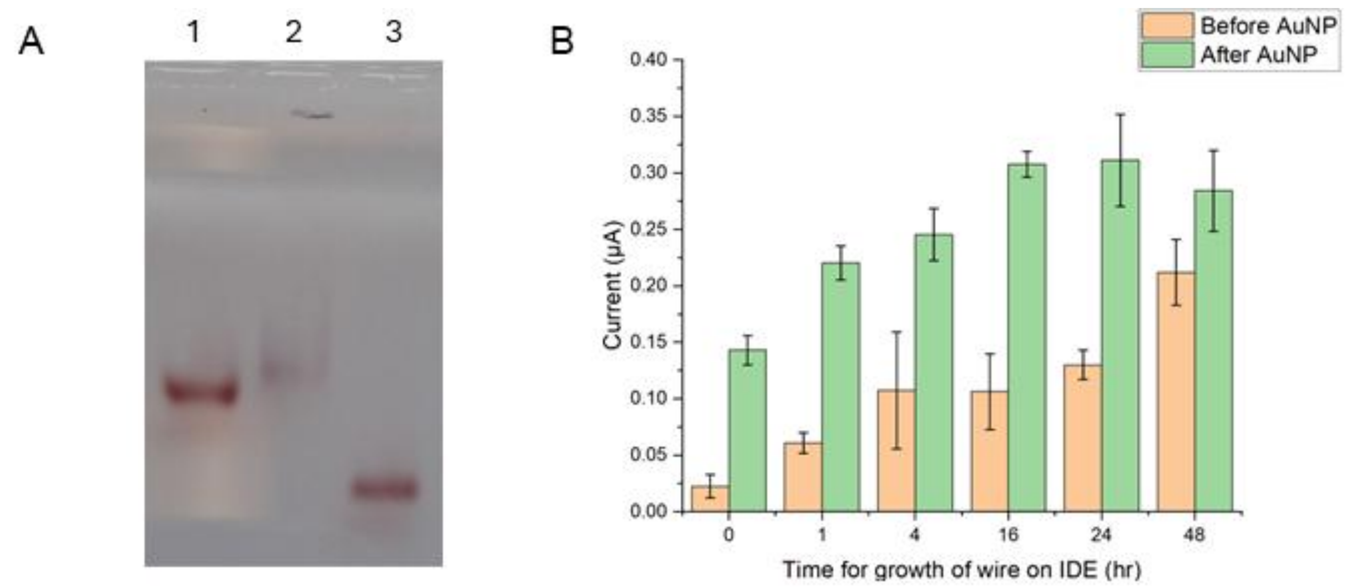

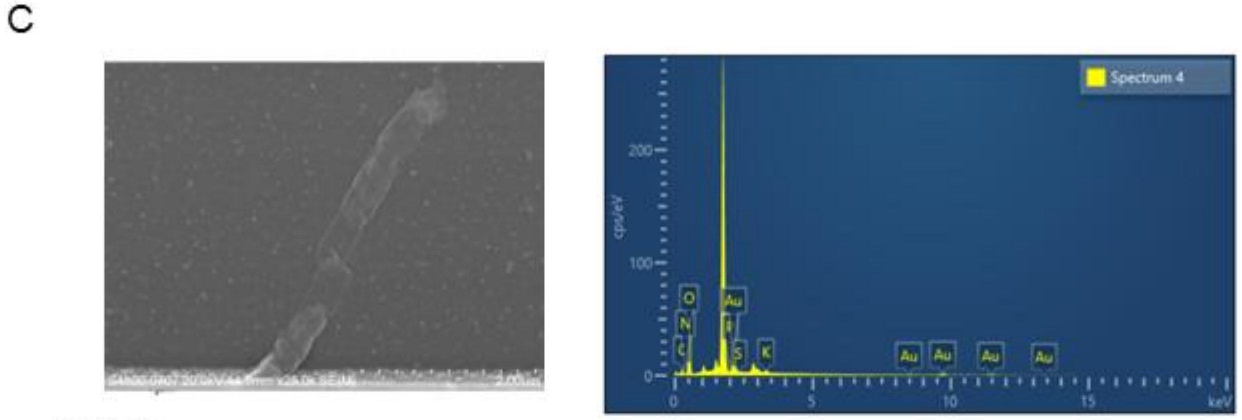

Quant Results:

Element	Line Type	Apparent Concentration	k Ratio	Wt%	Wt% Sigma	Standard Label	Factory Standard
C	K series	4.39	0.04393	12.83	0.96	C Vit	Yes
N	K series	0.91	0.00163	0.65	0.81	BN	Yes
O	K series	60.44	0.20338	56.82	0.97	SiO2	Yes
P	K series	3.92	0.02191	2.32	0.16	GaP	Yes
S	K series	0.65	0.00560	0.73	0.17	FeS2	Yes
K	K series	1.90	0.01611	1.73	0.14	KBr	Yes
Au	M series	26.13	0.26134	24.91	0.75	Au	Yes
Total:				100.00			

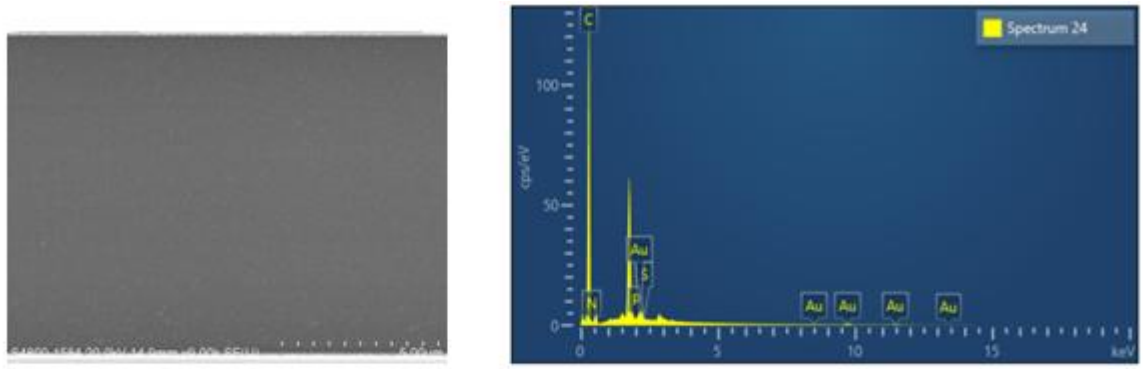

Quant Results:

Element	Line Type	Apparent Concentration	k Ratio	Wt%	Wt% Sigma	Standard Label	Factory Standard
C	K series	116.13	1.16129	84.69	0.79	C Vit	Yes
N	K series	6.06	0.01080	10.34	0.82	BN	Yes
P	K series	0.86	0.00481	0.31	0.03	GaP	Yes
S	K series	0.22	0.00192	0.13	0.03	FeS2	Yes
Au	M series	7.52	0.07519	4.52	0.15	Au	Yes
Total:				100.00			

Figure S19.Coating of gold nanoparticle on tesseract wire. (**A**) Agarose gel electrophoresis showing the conjugation between thiolated anchors and 10 nm gold nanoparticles. Lane 1, 10 nm gold nanoparticles in stock citrate buffer. Lane 2, 10 nm gold nanoparticles in 0.1 X PBS. Lane 3, DNA anchors conjugated 10 nm

gold nanoparticles in 0.1 X PBS. Faster migration was observed from the conjugate indicating presence of multiple negative charges. While aggregation was observed in the same buffer without DNA anchors. (**B**) Measurement of conductivity before and after coating gold nanoparticles on the tesseract wire at 0.8 V. (**C**) Validation of the presence of gold on the wire. Energy dispersive X-ray spectrum on the tesseract wire. Upper: The element gold was observed on the wire between electrodes on the IDE. Lower: IDE with zero-hour incubation time with tesseract cluster treated with the same protocol of coating gold nanoparticles.

Big Cube	
Bc1	GTTGGAACGGTCGTGGCACGCGTCGCCGTCATTCGCTCGGCGCCATGGTTAACGGGCGCCCTATGGGAGGCGGTGCCGTCACATGCCCTGCTCCTTGGTTAGGCCCGAATTTCGGCATGTCACGCGG
TA-Bc1	CGGGATTTTGGTATGTTGGAACGGTCGTGGCACGCGTCGCCGTCATTCGCTCGGCGCCATGGTTAACGGGCGCCCTATGGGAGGCGGTGCCGTCACATGCCCTGCTCCTTGGTTAGGCCCGAATTTCGGCATGTCACGCGG
TB-Bc1	GATTGGTGAAGCGTGTTGGAACGGTCGTGGCACGCGTCGCCGTCATTCGCTCGGCGCCATGGTTAACGGGCGCCCTATGGGAGGCGGTGCCGTCACATGCCCTGCTCCTTGGTTAGGCCCGAATTTCGGCATGTCACGCGG
Bc2	TGCCGAGGAATGAGCGCAGGTGTCGAACACTTGCTGCGCTCTAATTTCCGGAGTTAGCGCCACTTGACGGCGACGCGTGCCACGACCGTTCCAACTTTTGTGAGCTAAAACGGCCCGGGCTTGCATC
TA-Bc2	GGCCTCCTCCAAATTGCCGAGGAATGAGCGCAGGTGTCGAACACTTGCTGCGCTCTAATTTCCGGAGTTAGCGCCACTTGACGGCGACGCGTGCCACGACCGTTCCAACTTTTGTGAGCTAAAACGGCCCGGGCTTGCATC
TB-Bc2	TTGTTTGCGGATGGTGCCGAGGAATGAGCGCAGGTGTCGAACACTTGCTGCGCTCTAATTTCCGGAGTTAGCGCCACTTGACGGCGACGCGTGCCACGACCGTTCCAACTTTTGTGAGCTAAAACGGCCCGGGCTTGCATC
Bc3	AGGAGCAGGGCATGTGACGGCACCGCCTCCCTAGGGAAAGCCTGTGTACGTTACAGTCATCTGTGACCACACGATTCTGTGCTGGTCCCGGGCCGTGGCACATACCGACAAGCCATACCGCGCCACG
TA-Bc3	GCTGGATGTCACCGAGGAGCAGGGCATGTGACGGCACCGCCTCCCTAGGGAAAGCCTGTGTACGTTACAGTCATCTGTGACCACACGATTCTGTGCTGGTCCCGGGCCGTGGCACATACCGACAAGCCATACCGCGCCACG
TB-Bc3	CTTAGTTGATAAAAAGGAGCAGGGCATGTGACGGCACCGCCTCCCTAGGGAAAGCCTGTGTACGTTACAGTCATCTGTGACCACACGATTCTGTGCTGGTCCCGGGCCGTGGCACATACCGACAAGCCATACCGCGCCACG
Bc4	CGGCCCGGGACCAGCACAGAATCGTGTGGTCTGGATACTAATCGGTCAGGTGGGCCCGAGTGATAGTGTTCGACACCTGCGCTCATTCCTCGGCATCTTCAAAATTGGGCTTGAAGCTAGCTGTGTG

TA-Bc4	GCGATGGCGTGAGGCGGCCCGGGACCAGCACAGAATCGTGTGGTCTGGATACTAATCGGTCAGGTGGGCCCGAGTGATAGTGTTCGACACCTGCGCTCATTCCTCGGCATCTTCAAAATTGGGCTTGAAGCTAGCTGTGTG
TB-Bc4	GGAACATTATAATACGGCCCGGGACCAGCACAGAATCGTGTGGTCTGGATACTAATCGGTCAGGTGGGCCCGAGTGATAGTGTTCGACACCTGCGCTCATTCCTCGGCATCTTCAAAATTGGGCTTGAAGCTAGCTGTGTG

Small Cube	
Sc1	ACACCCTCCTAGCACCCGCTGTGCGTCGATCGGGTAATA
Sc2	CTGGCCTTCTCAGAACTCTTAGCACGGATTGCGGGTGCT
Sc3	TGAGGGAACTTATTACCCGTCCAAATGCGTAGAGTCCCG
Sc4	CCAGCCTGGTCGGGACTCTTAGGAGAGCGTAGAGTTCTG
Sc1-Cy5	Cy5-ACACCCTCCTAGCACCCGCTGTGCGTCGATCGGGTAATA
Sc2-Cy3	Cy3-CTGGCCTTCTCAGAACTCTTAGCACGGATTGCGGGTGCT
Sc3-Cy3	Cy3-TGAGGGAACTTATTACCCGTCCAAATGCGTAGAGTCCCG
Sc4-Cy5	Cy5-CCAGCCTGGTCGGGACTCTTAGGAGAGCGTAGAGTTCTG
Trapezoidal Prism A	
A1	TAGGGCGCCCGTTAACCATGGCGCCGAGCGATGGGCAAAAAAGCCTGTTCCCTCATGAATGGGAACGGT
TA-A1	CCATCCGCAAACAATAGGGCGCCCGTTAACCATGGCGCCGAGCGATGGGCAAAAAAGCCTGTTCCCTCATGAATGGGAACGGT
TB-A1	ATTTGGAGGAGGCCTAGGGCGCCCGTTAACCATGGCGCCGAGCGATGGGCAAAAAAGCCTGTTCCCTCATGAATGGGAACGGT
A2	TCACTCGGGCCCACCTGACCGATTAGTATCCTAGGAGAGCCGGTGTGAAGGCCAGTCACGGCGCCGTAA

TA-A2	TTTTATCAACTAAGTCACTCGGGCCCACCTGACCGATTAGTATCCTAGGAGAGCCGGTGTGAAGGCCAGTCACGGCGCCGTAA
TB-A2	CGGTGACATCCAGCTCACTCGGGCCCACCTGACCGATTAGTATCCTAGGAGAGCCGGTGTGAAGGCCAGTCACGGCGCCGTAA
A3	CAGATGACTGTAACGTACACAGGCTTTCCCTTACCGTTCCCATTCTCCAGGCTGGTCACCGGCTCTCCT
TA-A3	ACGCTTCACCAATCCAGATGACTGTAACGTACACAGGCTTTCCCTTACCGTTCCCATTCTCCAGGCTGGTCACCGGCTCTCCT
TB-A3	ATACCAAAATCCCGCAGATGACTGTAACGTACACAGGCTTTCCCTTACCGTTCCCATTCTCCAGGCTGGTCACCGGCTCTCCT
A4	GTGGCGCTAACTCCGGAAATTAGAGCGCAGCTTTACGGCGCCGTGTGGAGGGTGTTGGCTTTTTTGCCC
TA-A4	TATTATAATGTTCCGTGGCGCTAACTCCGGAAATTAGAGCGCAGCTTTACGGCGCCGTGTGGAGGGTGTTGGCTTTTTTGCCC
TB-A4	CCTCACGCCATCGCGTGGCGCTAACTCCGGAAATTAGAGCGCAGCTTTACGGCGCCGTGTGGAGGGTGTTGGCTTTTTTGCCC
Trapezoidal Prism B	
B1	ACGGGCCGGACTCTCCGCGTGACATGCCGAAATTCGGGCCTAACCTGGGCCGGTGCCCTTCGCATTTGG
B2	ACGTCGGCGTGGCTCACACAGCTAGCTTCAAGCCCAATTTTGAAGTACGATAGTAGAGCTATCCGTGCT
B3	AGGGCACCGGCCCTCGTGGCGCGGTATGGCTTGTCGGTATGTGCCTGCCACGCCGACGTTCGCTCTCCT
B4	GCTCTACTATCGTTGATGCAAGCCCGGGCCGTTTTAGCTCACAAATGAGTCCGGCCCGTTTCGACGCAC
Anchors on interdigitated electrode	
TB-A2c	GCTGGATGTCACCG-HS-SH

TB-A4c	GCGATGGCGTGAGG-HS-SH
TB-Bc1c	ACGCTTCACCAATC-HS-SH
TB-Bc2c	CCATCCGCAAACAA-HS-SH
TA-Bc3c	CGGTGACATCCAGC-HS-SH
TA-Bc4c	CCTCACGCCATCGC-HS-SH
TA-A3c	GATTGGTGAAGCGT-HS-SH
TA-A1c	TTGTTTGCGGATGG-HS-SH
Tetrahedron A	
TetraA1	GCTGGATGTCACCGCGGACGGCGGATGCATAGCATCGATACCGTTCAGCCCTTGGAGATA ACCTGTAACCTGGTGTACACACCCATCCATGATCCACCTATTAGAG
TetraA2	ATACCAAAATCCCGCGATCCTCATGGCCTCGTGGCCATGAGGCGTAGGGTACGACCCACC AGACGCGCATGTAGCTCACCAGGTTACAGGTTATCTCCAAGGGCTG
TetraA3	ATTTGGAGGAGGCCCGCCTCATGGCCACGAGGCCATGAGGATCGTACGGTATCGATGCTA TGCATCCGCCGTCCGTCTAGACCCGTCGCCACGCGTCCAACCTGGC
TetraA4	CCTCACGCCATCGCGCTACATGCGCGTCTGGTGGGTCGTACCCTTGCCAGGTTGGACGCG TGGCGACGGGTCTAGTCTCTAATAGGTGGATCATGGATGGGTGTGT

Tetrahedron B	
TetraB1	CGGTGACATCCAGCAGTGGAGGGAGGAACATACTTACACGTCACTTCGCAGGCCTACATT GTGTGTTACACGCAGTGGCTACCCAATCGACTCCCCAGTTGTAGAG
TetraB2	GCGATGGCGTGAGGGTTGGGCGCTTACGACTTCGCGAACGGCGGTTGTGGAACCAGATC GGACTCATACAGCAGCTCTGCGTGTAACACACAATGTAGGCCTGCGA
TetraB3	GGCCTCCTCCAAATCCGCCGTTCGCGAAGTCGTAAGCGCCCAACTGTGACGTGTAAGTAT GTTCCTCCCTCCACTTATCACTGCCCGCATACTCTAAGCCACGAAG
TetraB4	CGGGATTTTGGTATGCTGCTGTATGAGTCCGATCTGGTTCCACATCTTCGTGGCTTAGAGTA TGCGGGCAGTGATTCTCTACAACTGGGGAGTCGATTGGGTAGCC

Fluorescent anchors for confocal microscopy	
TA-11 compleCy3	Cy3-GGAACATTATAATA
Duplex Control	
Cy5 control-3'	GTGGCTGAAACTCTCCCCGTGCGTGTTCG-Cy5
Cy3 control-5'	Cy3-ACGGGGAGAGTTTCAGCCAC
Cy3 control-9bp	CGAACACGC

Table S1. List of oligonucleotides used in this work.
